# *Candida albicans* induces mucosal bacterial dysbiosis that promotes invasive infection

**DOI:** 10.1371/journal.ppat.1007717

**Published:** 2019-04-22

**Authors:** Martinna Bertolini, Amit Ranjan, Angela Thompson, Patricia I. Diaz, Takanori Sobue, Kendra Maas, Anna Dongari-Bagtzoglou

**Affiliations:** 1 Department of Oral Health Sciences, University of Connecticut Health Center, Farmington, Connecticut, United States of America; 2 Microbial Analysis, Resources, and Services Core, University of Connecticut, Storrs, Connecticut, United States of America; Louisiana State University Health Sciences Center New Orleans, UNITED STATES

## Abstract

Infectious complications are a common cause of morbidity and mortality in cancer patients undergoing chemotherapy due to increased risk of oral and gastrointestinal candidiasis, candidemia and septicemia. Interactions between *C*. *albicans* and endogenous mucosal bacteria are important in understanding the mechanisms of invasive infection. We published a mouse intravenous chemotherapy model that recapitulates oral and intestinal mucositis, and myelosuppression in patients receiving 5-fluorouracil. We used this model to study the influence of *C*. *albicans* on the mucosal bacterial microbiome and compared global community changes in the oral and intestinal mucosa of the same mice. We validated 16S rRNA gene sequencing data by qPCR, *in situ* hybridization and culture approaches. Mice receiving both 5Fu and *C*. *albicans* had an endogenous bacterial overgrowth on the oral but not the small intestinal mucosa. *C*. *albicans* infection was associated with loss of mucosal bacterial diversity in both sites with indigenous *Stenotrophomonas*, *Alphaproteobacteria* and *Enterococcus* species dominating the small intestinal, and *Enterococcus* species dominating the oral mucosa. Both immunosuppression and *Candida* infection contributed to changes in the oral microbiota. Enterococci isolated from mice with oropharyngeal candidiasis were implicated in degrading the epithelial junction protein E-cadherin and increasing the permeability of the oral epithelial barrier *in vitro*. Importantly, depletion of these organisms with antibiotics in vivo attenuated oral mucosal E-cadherin degradation and *C*. *albicans* invasion without affecting fungal burdens, indicating that bacterial community changes represent overt dysbiosis. Our studies demonstrate a complex interaction between *C*. *albicans*, the resident mucosal bacterial microbiota and the host environment in pathogenesis. We shed significant new light on the role of *C*. *albicans* in shaping resident bacterial communities and driving mucosal dysbiosis.

## Author summary

In cancer patients receiving high dose chemotherapy mucosal candidiasis is common and can lead to invasive, systemic fungal infection with mortality ranging 25–30%. We showed that in chemotherapy-immunosuppressed mice *C*. *albicans* induces a dysbiotic switch that favors growth of enterococci on mucosal surfaces, particularly on the oral mucosa. Overgrowth of these organisms led to enhanced oral mucosal barrier breach by *C*. *albicans*. Like *C*. *albicans*, *Enterococcus* species are a major concern in critical care patients due to their involvement in sepsis and resistance to multiple antibiotics. There is mounting evidence that *C*. *albicans* and *Enterococci* co-exist in human disease samples. Our discoveries provide the first experimental evidence of pathogenic synergy between these microorganisms in the context of dysbiosis.

## Introduction

Oropharyngeal (OPC) and gastrointestinal candidiasis are common infections in patients on high dose cancer chemotherapy, mostly attributed to *Candida albicans*. In these populations prevalence rates of OPC range between 25–40% [1]. Cytotoxic chemotherapy also causes an inflammatory form of oral and gastrointestinal injury known as mucositis [2]. Mucosal injury combined with the myelosuppressive effects of chemotherapy, promote bacterial and fungal translocation through mucosal barriers leading to bloodstream infections, a major cause of morbidity and mortality in this patient population [3,4]. There is some evidence that the oral and intestinal bacterial microbiota in humans may be altered by cytotoxic chemotherapy, although the effects on the fungal microbiota are less clear [5,6].

In a healthy host, unperturbed resident commensal bacterial communities play an important role in limiting *C*. *albicans* colonization in mucosal sites [7]. However, when the microbial equilibrium is changed by immunosuppression certain bacterial species may overgrow and form mutualistic relationships with *C*. *albicans*. This in turn may lead to a well-coordinated dysbiosis which amplifies mucosal damage. We recently revealed mutualistic relationships between *C*. *albicans* and commensal oral streptococci in a mouse model of OPC [8,9,10,11]. However, these studies were performed with bacteria that are not part of the indigenous mouse microbiota. A role for indigenous bacterial community-mediated dysbiosis in fungal pathogenesis has never been examined. Furthermore, the effects of *C*. *albicans* in modulating commensal bacterial community composition have only been studied in the mouse gastric and intestinal mucosa. In these studies *C*. *albicans* was shown to favor growth of endogenous enterococci post-antibiotics treatment [12,13,14]. Studies focusing on the interplay between *C*. *albicans* and resident oral mucosa bacteria in health and disease are nonexistent.

Chemotherapy-associated mucosal candidiasis studies use high doses of chemotherapeutic agents and focus exclusively on the development of gastrointestinal or disseminated candidiasis. The vast majority of these studies also used antibiotics aimed at increasing gastrointestinal fungal burdens [15,16,17,18]. We recently developed a mouse model of low dose intravenous 5Fu administration that recapitulates the histopathologic changes associated with cancer chemotherapy-induced mucositis [19]. Using this model we tested the hypothesis that mucosal injury combined with peripheral neutropenia induced by 5Fu, are sufficient for the development of oropharyngeal and intestinal candidiasis in mice with unperturbed resident bacterial microbiota. For the first time we also examined the influence of 5Fu and candidiasis on the mucosal bacterial microbiome and the reciprocal effect of the resident microbiota on *C*. *albicans* virulence.

## Results

### 5Fu predisposed mice to oropharyngeal and intestinal candidiasis, increased oral bacterial burdens and disseminated infection

**Fig 1:**
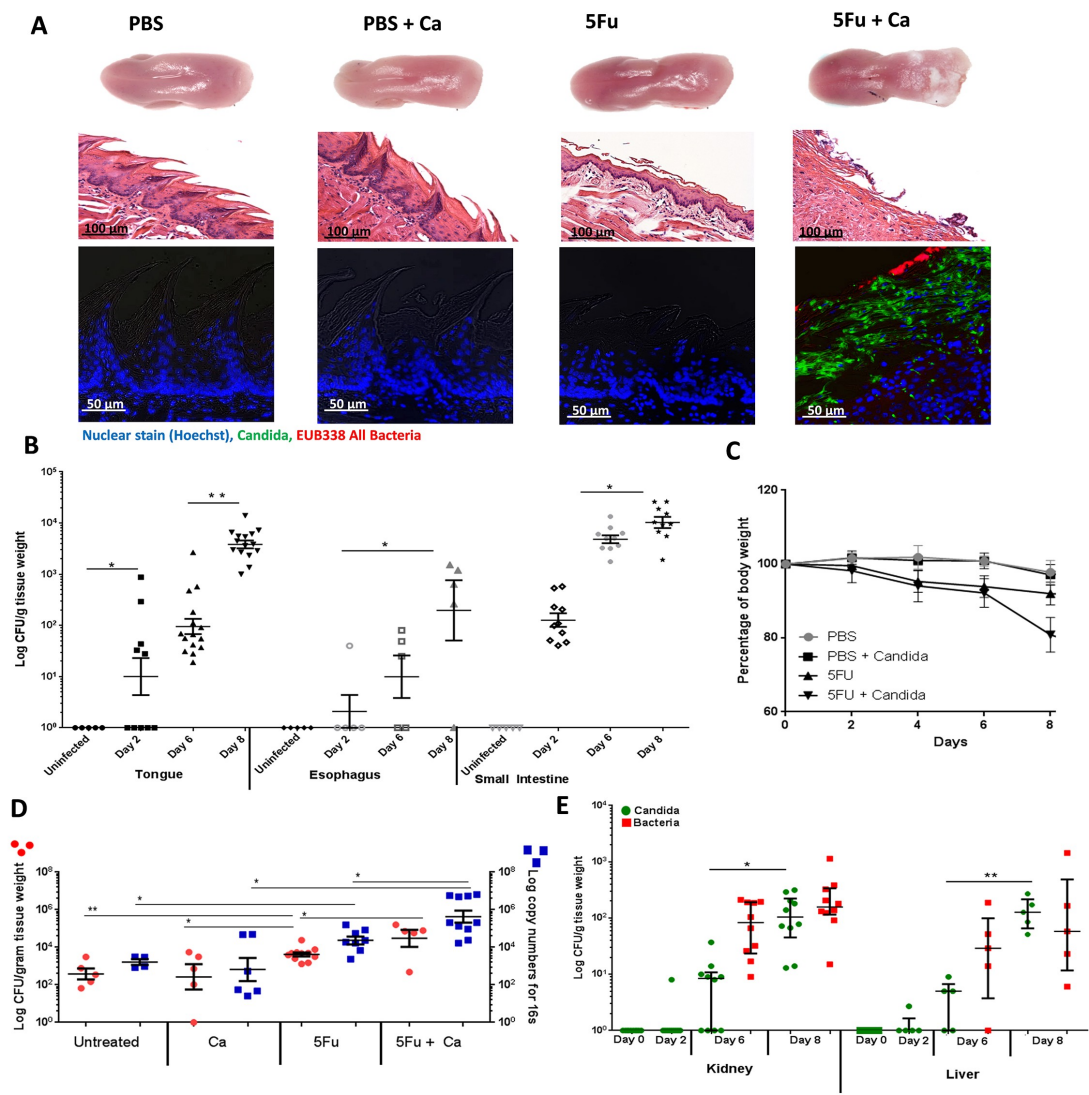
Effect of 5Fu administration on *Candida albicans* and endogenous bacterial burdens. A: Mucosal biofilm forming on tongues excised 8 days post-treatment. The group receiving 5Fu (50mg/kg, IV, every 48 hours) + *C*. *albicans* SC5314 (5Fu+Ca) had a thick mucosal biofilm covering the posterior tongue surface. Middle panel includes H&E-stains showing reduced epithelial thickness, papillary atrophy, desquamation and erosion in the 5Fu group and deep epithelial ulcerations in the 5Fu+Ca group. Lower panel shows immunofluorescence combined with fluorescence in situ hybridization (immuno-FISH) to simultaneously visualize *C*. *albicans* and bacterial commensals in tongue biofilms. *C*. *albicans* stained with a polyclonal anti-*Candida* antibody (green), commensal bacteria stained with EUB338-Alexa 546 probe (red) and cell nuclei counterstained with Hoechst 33258 (blue). B: Recovery of *C*. *albicans* from tongues, esophagus and small intestines in control untreated mice, and in mice receiving 5Fu every 48h with *C*. *albicans* SC5314 added daily in the drinking water. Shown are colony-forming unit (CFU) counts from organs harvested at baseline (uninfected), then 2, 6 and 8 days after the first 5Fu injection. Tissues were weighed, homogenized, serially diluted and plated for counts on Sabouraud Dextrose Agar containing chloramphenicol. Similar counts of *C*. *albicans* were obtained in CHROMagar Candida media with no other species identified (not shown). CFU assays showed that fungal burdens increased significantly over time in all tissues. Log CFU counts/gm of tissue are shown from 2 independent mouse experiments, with 5–15 mice per group; bars represent means ± SEM. *p<0.05, **p<0.0001. C: Body weight loss during the eight-day experimental period, expressed as percentage of initial weight (day 0) in 5–10 animals per group from 1–2 independent experiments. Error bars represent SEM. Mice receiving 5Fu every 48h lost weight slowly over time, while mice receiving 5Fu every 48 hours with *C*. *albicans* SC5314 added daily in the drinking water reached 20% body of total weight loss by day 8 (p<0.05 for a comparison with the 5Fu group for d8). D: Tongue bacterial loads compared among untreated mice, mice inoculated with *C*. *albicans* SC5314 (Ca), mice receiving 5Fu alone, and mice receiving both 5Fu and *C*. *albicans* SC5314 (5Fu+Ca) daily in the drinking water. Mice were sacrificed 8 days later. Tongues were weighed, homogenized, serially diluted and plated and results expressed as CFU counts/gm of tissue (left Y-axis, red dots). The total bacterial biomass (log 16S rRNA gene copy numbers/gm of tissue) was also quantified by real-time qPCR (right Y-axis, blue squares). Both assays showed a significant increase in endogenous tongue bacteria in 5Fu treated mice when compared to untreated and a further increase in the 5Fu + *C*. *albicans* group, which was significantly higher than the 5Fu group (p = 0.008). Data shown are from 1–2 independent mouse experiments, with 4–10 mice per group; bars represent means ± SEM. *p<0.01, p**<0.005, ***p<0.0005. E: Recovery of *C*. *albicans* and endogenous bacteria from kidneys and livers in mice receiving 5Fu and *C*. *albicans* SC5314 daily in the drinking water. Mice were sacrificed on days indicated. Kidneys and livers were weighed, homogenized, serially diluted and plated for CFU counts. Results show a time-dependent increase in fungal and bacterial dissemination to both organs. Log CFU counts/gm of tissue are shown from 2 independent mouse experiments, with 5–10 mice per group; bars represent means ± SEM. *p<0.0001, **p<0.0005.

In the first series of experiments we tested whether intravenous, low dose 5Fu administration increases the susceptibility of mice to oral and intestinal candidiasis. Fig 1A shows that mice receiving four doses of 5Fu (50 mg/kg every other day) developed tongue papillary atrophy, epithelial desquamation and erosion, consistent with early stages of mucositis [19,20]. While this treatment caused gradual depletion of mature neutrophils in the bone marrow and blood, a steady infiltrate of CD11b^+^/LyG^+^ cells was found in the tongue mucosa reflecting local inflammation secondary to mucosal injury (S1A, S1B and S1C Fig). Mice receiving 5Fu+*C*. *albicans* developed thick white biofilms covering the posterior tongue surface, associated with epithelial ulcerations (Fig 1A). In these mice *C*. *albicans* fungal burdens increased significantly over time in all mucosal surfaces (Fig 1B). This group also lost significantly more weight than mice treated with 5Fu alone (Fig 1C). We also noted almost complete absence of neutrophils in the tongues of this group (S1A, S1B and S1C Fig). This was not due to absence of neutrophil activating cytokines since KC and IL-6 were significantly increased in infected tissues (S1D Fig).

In mice treated with 5Fu and infected with *C*. *albicans*, tongue biofilms were composed by *C*. *albicans* and indigenous bacteria (Fig 1A, lower panel). The PBS control group receiving *C*. *albicans* showed complete absence of biofilms and pathology and an early increase in tongue neutrophils consistent with other reports (Figs 1A and S1B) [21]. No bacterial biofilms were seen on the surface of 5Fu-only or *C*. *albicans*-only treated mice using a pan-eubacterial FISH probe (Fig 1A, lower panel).

The presence of endogenous bacteria in biofilms with *C*. *albicans* prompted a closer examination of the resident bacterial microbiota. We first compared viable (CFU) and total (qPCR) bacterial biomass across all experimental groups at the end of the infection period. As seen in Fig 1D there was a significant increase in the viable and total bacterial biomass in mice treated with 5Fu and infected with *C*. *albicans*, compared to all other groups. We also noted a significant increase in endogenous bacteria with 5Fu treatment alone, compared to untreated control and *C*. *albicans* alone groups. However, since the increase in bacterial biomass with 5Fu did not lead to the development of biofilms (Fig 1A), this suggested that in the absence of *C*. *albicans* bacteria could not organize in biofilm community structures on the tongue surface.

A positive correlation was found in fungal and bacterial loads on the same tongues at the end of the experimental period (S2A Fig), suggesting that fungal burdens increased in concert with endogenous bacterial burdens. To confirm this we examined time-dependent changes in the viable and total bacterial biomass on the tongue mucosa of mice in this group. S2B Fig shows a gradual increase in bacterial biomass over time, confirming that endogenous bacteria increased as *C*. *albicans* burdens rose (Fig 1B). Collectively these data show that *C*. *albicans* infection in 5Fu-treated mice promotes bacterial overgrowth on the oral mucosa and that 5Fu treatment contributes to this effect.

In contrast, mucosa-associated bacterial loads did not change significantly after 8 days of infection in the jejunum of mice with candidiasis (S2C Fig), suggesting that the effects of *C*. *albicans* infection on commensal bacteria are mucosal site-specific. Strain SC5314 colonized the jejunum of PBS control mice in low numbers (not shown) and this was associated with a significant reduction in resident bacterial CFUs (S2D Fig). A smaller decrease in the viable bacterial biomass was also noted in mice receiving both 5Fu and *C*. *albicans*. These results suggested that growth of *C*. *albicans* in this mucosal site displaces endogenous bacterial communities.

Cytotoxic chemotherapy elevates the risk for bloodstream infections [22], we thus asked whether *C*. *albicans* disseminated in distant organs. We found a time-dependent increase in fungal burdens in kidneys and livers, accompanied by increased bacterial burdens in the same organs (Fig 1E). Collectively these data suggest that 5Fu creates favorable conditions for *C*. *albicans* and endogenous bacterial growth in the upper and lower alimentary tract mucosae and systemic dissemination of bacteria and fungi. Importantly, mice receiving both 5Fu and *C*. *albicans* had an endogenous bacterial overgrowth in the oral mucosa that exceeded 5Fu treatment alone.

### In 5Fu-treated mice OPC was associated with loss of bacterial diversity and indigenous enterococcal bloom

**Fig 2:**
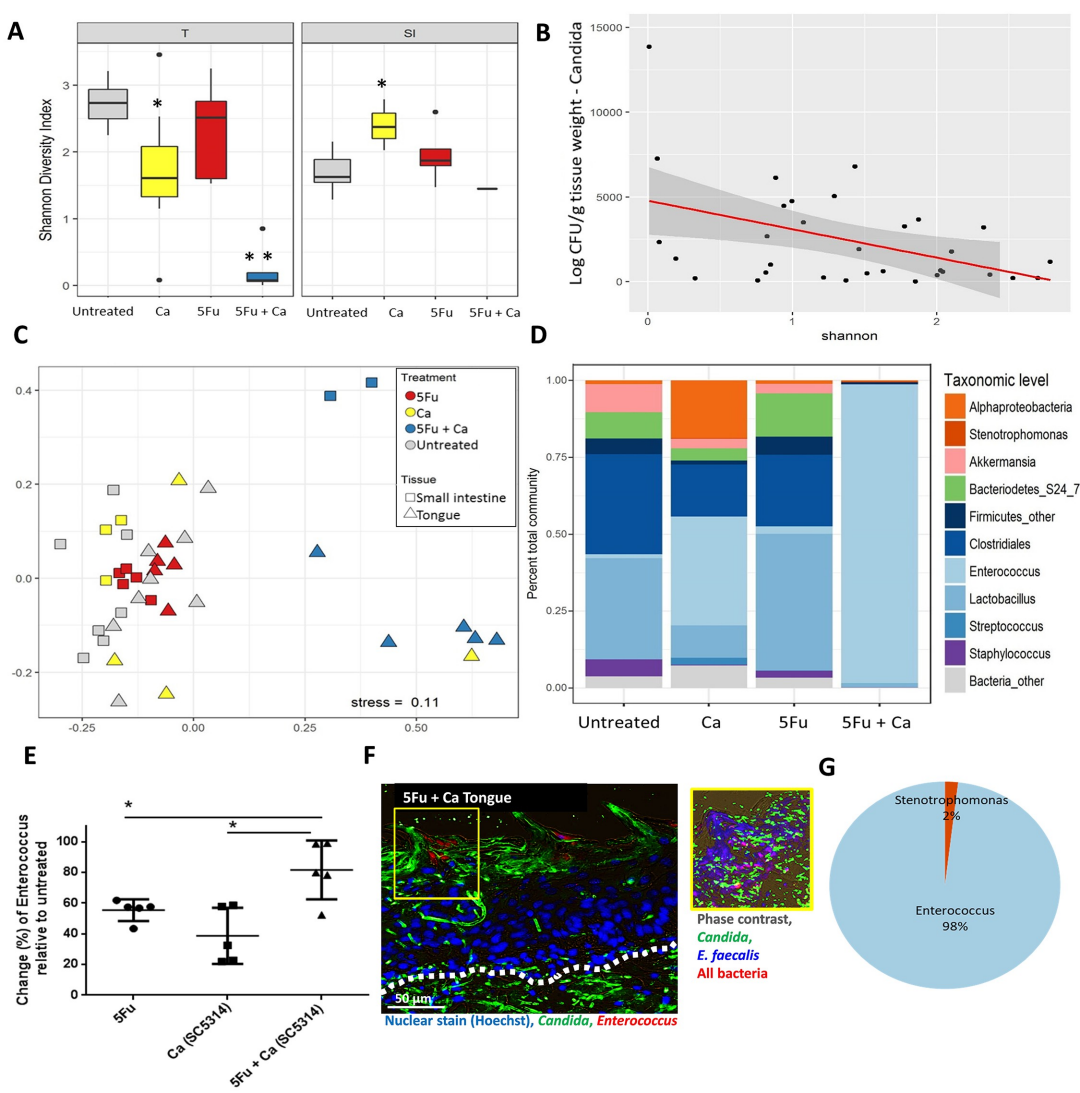
Mucosa-associated bacterial microbiota profiling. A: Microbial DNA was extracted from tongues (T) and small intestines (SI) of the same mice on day 8. The V4 hypervariable region of the 16S rDNA gene was amplified and sequenced. Box plot showing Shannon Diversity Index in untreated mice, mice receiving *C*. *albicans* SC5314 daily in the drinking water (Ca), mice receiving 5Fu alone and a combination of the two (5Fu+Ca). Mean diversity index values are shown from 5 mice in each group. In mice inoculated with *C*. *albicans* alone (Ca) bacterial diversity decreased in the tongue but increased in the small intestine mucosa (*p<0.05 for a comparison to untreated groups). For mice receiving 5Fu and *C*. *albicans* (5Fu+Ca) there was a significant further reduction in bacterial diversity in tongue but not small intestinal tissues (**p<0.001). B: Linear regression analysis of *C*. *albicans* CFUs plotted against the Shannon Diversity Index of the bacterial communities in the same tongues. Data include all mice receiving *C*. *albicans* SC5314 with or without chemotherapy, at all time points (5 mice/group, t = 2, 6 or 8 days, n = 30). This analysis showed higher *C*. *albicans* burdens correlating with lower bacterial diversity (R^2^ = 0.17, p<0.05). C: Beta diversity assessed by nonmetric multidimensional scaling (NMS) based on Bray-Curtis dissimilarities among four treatment groups. Shown are community structures in the untreated control, *C*. *albicans* alone, and the two chemotherapy groups (Untreated: grey, Ca: yellow 5Fu: red, 5Fu+Ca: blue; n = 5 mice/group) in tongues (triangles) and small intestines (squares) of the same mice. Results represent community structure differences at the end of the experimental period (day 8). Microbial communities clustered by type of treatment, indicating a significant effect of *C*. *albicans* in 5Fu treated mice. Samples in this group also clustered by site, indicating that the two sites harbor microbiomes with distinct global community structures and composition by day 8. The type of treatment explained 16% of the variability (p<0.01), whereas the site explained 8.5% of the variability (p<0.01) among samples. D: Mean relative abundance of OTU sequences assigned to one of the top 10% prominent taxa identified on mouse tongues, in each of the four treatment groups, at the end of the experimental period (day 8, n = 5 mice/group). *Enterococcus* was the most abundant taxon in both *C*. *albicans*-inoculated groups. However, relative abundance of this genus was more extensive in mice that also received chemotherapy, in which they represented ~98% of the total bacterial community. E: Validation of the bacterial microbiome sequencing data by qPCR. Genus level quantification was performed for *Enterococcus* on tongue tissues by qPCR. Data represent change in percentage of *Enterococcus* load in 5Fu, Ca (*C*. *albicans* SC5314) and 5Fu + Ca (SC5314) groups compared to untreated control mice. Mice receiving both 5Fu and *C*. *albicans* had significantly higher *Enterococcus* increase when compared to all other groups. Results are shown from 5 mice per group; bars represent means ± SEM. *p<0.001. F: Tongue tissue section from mice receiving 5Fu and *C*. *albicans* SC5314 for 8 days. Tissue sections were stained by immuno-FISH to simultaneously visualize *C*. *albicans* and endogenous bacteria. Left panel shows staining with the *Enterococcus/Lactobacillus* specific probe LAB158 (red), anti-*Candida*-FITC polyclonal antibody (green) and cell nuclei counterstained with Hoechst 33258 (blue). Note *Candida* invasion in the submucosal compartment (bellow the white dotted line). On the right panel immuno-FISH staining of a serial section from the same tissue is shown representing the biofilm area in yellow. Section was triple-stained with the all bacteria probe EUB338 (red), the *Enterococcus faecalis* specific probe ENFL84 (blue), and anti-*Candida* antibody (green). Note the almost complete overlap of the blue and red signals resulting in blue-purple staining bacteria representing *E*. *faecalis*. G: Identification of bacteria isolated from the tongues of mice after 8 days of receiving 5Fu and *C*. *albicans* SC5314. The V4 variable region of the 16S rDNA gene was amplified and sequenced by Illumina. 98% of the OTU sequences from five isolates aligned with the genus *Enterococcus*. All isolates were identified as *E*. *faecalis* with species-specific primers and PCR.

The increase in oral bacterial burdens observed in mice with OPC raised the possibility of global mucosa-associated bacterial microbiota changes. We thus performed high throughput sequencing of the V4 hypervariable region of the 16S rRNA gene in DNA extracted from tongues. We also analyzed the bacterial microbiome of the jejunum in the same mice, for comparison. To explore differences in bacterial community composition within each treatment and control groups we first analyzed alpha diversity as reflected by Shannon index (Fig 2A) and richness estimates (S3A Fig). Compared to the untreated group the 5Fu group was not significantly different in community diversity when either the tongue or jejunum were examined. However, inoculation with *C*. *albicans* alone induced a decrease in bacterial diversity in the oral mucosa, whereas diversity in the jejunum increased (Fig 2A), consistent with other reports [23]. This illustrates that daily inoculation with this strain had an impact on oral biodiversity even though colonization was below the sensitivity limit of the CFU assay. A dramatic drop in bacterial diversity was noted in the tongues of mice receiving *C*. *albicans* and 5Fu (Fig 2A), consistent with the reduction in the number of species (OTUs) observed (S3A Fig). A significant reduction in the average number of species was also observed in the jejunum of the same mice (S3A Fig). To further explore the impact of *C*. *albicans* on oral bacterial diversity we performed regression analysis of the Shannon index in relation to *C*. *albicans* CFUs in the same tongues. This analysis showed a negative correlation between oral fungal burdens and bacterial diversity (Fig 2B). In summary, these data show that in a disease-permissive host environment *C*. *albicans* reduces the bacterial diversity both in the upper and lower GI tract mucosa.

To better understand the effect of *C*. *albicans* in chemotherapy-treated mice we performed beta diversity analyses comparing the 5Fu and 5Fu+*C*. *albicans* groups. Non-metric multidimensional scaling (NMS) analysis of Bray-Curtis dissimilarities between the two treatments showed that the bacterial microbiome composition was distinct in mice receiving 5Fu compared to mice receiving 5Fu+*C*. *albicans* in both sites (Fig 2C). Community structure from most tongues of the group receiving *C*. *albicans* alone, clustered closer to the two uninfected control groups, suggesting that *C*. *albicans* alone does not alter the composition of the oral microbiome significantly. As expected, microbial communities also clustered by site indicating that they harbor microbiota with distinct global community structures and composition. Time-dependent analysis of beta diversity changes in the microbiomes of the two treatment groups further showed that *C*. *albicans* caused a profound disruption of the tongue and small intestinal community structure after 6 days of chemotherapy (S3B Fig).

Analysis of the top 10% prevalent bacterial OTUs on the tongue mucosa revealed distinct genus level differences among the four experimental groups (Fig 2D). Specifically, an increase in the relative abundance of the genus *Enterococcus* was noted on the oral mucosa in both groups receiving *C*. *albicans*, with the most dramatic increase in mice also receiving 5Fu (representing ~99% of all OTUs), explaining the almost complete loss of diversity in this group (Fig 2A). A smaller increase in the enterococcal OTUs was also noted with 5Fu treatment alone (Fig 2D). The increase in enterococcal biomass in all groups over untreated control, with the 5Fu+*C*. *albicans* group being the highest, was validated by a genus-specific qPCR assay (Fig 2E). Importantly, endogenous enterococci were identified in mixed tongue biofilms with *C*. *albicans* using genus and species (*E*. *faecalis*)-specific FISH probes. In these biofilms *C*. *albicans* was noted invading into the submucosal tongue compartment (Fig 2F, demarcated area). Furthermore, 98% of the OTUs in bacterial cultures from these tongues were identified as enterococci by 16S rRNA gene sequencing (Fig 2G) and all isolates PCR-amplified with *E*. *faecalis*-specific primers (not shown).

A similar analysis of the most abundant OTUs in the jejunum mucosa of the same mice showed the most dominant taxa to be *Stenotrophomonas*, *Alphaproteobacteria* and to a lesser extent *Enterococcus* (S3C Fig). A reduction in the relative abundance of *Lactobacillus* species was observed in both the tongue and intestinal mucosa of infected mice (Figs 2D and S3C). This further shows that despite the distinct mucosal microbiota shifts in the two sites *C*. *albicans* infection led to *Enterococcus* growth and *Lactobacillus* reduction in both sites.

### Both *C*. *albicans* and 5Fu contributed to changes in oral mucosal bacterial burdens and biodiversity

**Fig 3:**
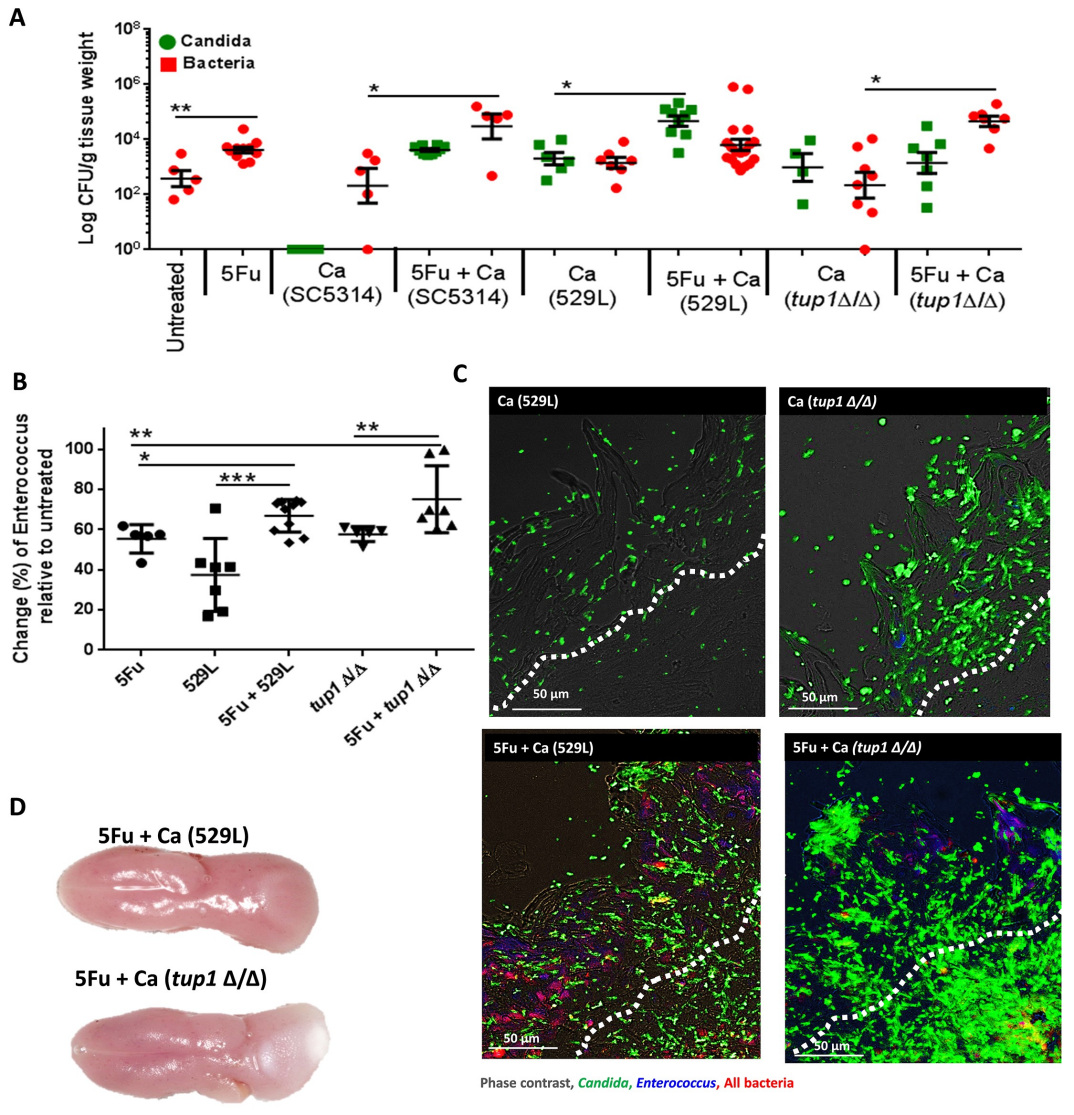
Effects of different *C*. *albicans* strains and 5Fu on oral bacteria. A: Recovery of *C*. *albicans* (green) and total cultivable bacteria (red) from tongues of untreated mice, mice receiving 5Fu, mice receiving *C*. *albicans* (Ca) and a combination (5Fu+Ca). *C*. *albicans* strains SC5314, 529L or the *tup1Δ/Δ* deletion mutant were used in these experiments. Tongues were weighed, homogenized, serially diluted and plated for CFU counts. Strains 529L and *tup1Δ/Δ* were able to stably colonize the tongue mucosa in the absence of 5Fu, however, this was not associated with increased oral bacterial loads. On the other hand the combination of 5Fu with both strains led to increased bacterial loads. Log CFU counts/gm of tissue are shown from 2 independent mouse experiments, with 4–8 mice per group; bars represent means ± SEM. *p<0.01, **p<0.0001. B: Tongue-associated *Enterococcus* biomass as assessed by genus-level qPCR. Data represent percentage change of *Enterococcus* genome copy numbers relative to untreated mice. Groups included untreated mice, mice receiving 5Fu, strain 529L alone, strain 529L + 5Fu, the *tup1Δ/Δ* deletion mutant alone and the *tup1Δ/Δ* deletion mutant + 5Fu. All treatments led to an increase in the enterococcal biomass, however mice receiving both 5Fu and *C*. *albicans* had the highest increase. Results shown are from 5–8 mice per group in two experiments; bars represent means ± SEM. *p<0.05, **p<0.005, ***p<0.002. C: Tongue tissue sections from mice receiving *C*. *albicans* 529L or *tup1Δ/Δ* deletion mutant in the absence (left panel) or presence (right panel) of concomitant 5Fu chemotherapy for 8 days. Tissue sections were triple-stained with the all bacteria probe EUB338 (red), the *E*. *faecalis* specific probe ENFL84 (blue), and anti-*Candida* antibody (green). Enterococcal signal shown as purple-blue is clearly visible in mice receiving 5Fu with both strains. In these mice there was fungal invasion into the oral submucosa (bellow the white dotted line), which was more pronounced with the *tup1Δ/Δ* mutant. D: Representative tongues excised from mice receiving 5Fu with strain 529L (top) or the *tup1Δ/Δ* deletion mutant (bottom), 8 days after infection. Note the white biofilm area in the posterior surface with the *tup1Δ/Δ* deletion mutant.

*C*. *albicans* SC5314 does not stably colonize the oral mucosa of healthy mice when inoculated via the drinking water and viable counts are below the sensitivity of the CFU assay (Fig 3A). In order to further explore the relative contributions of 5Fu treatment and *C*. *albicans* in oral bacterial changes we asked whether strains that stably colonize the oral mucosa of healthy mice could induce similar bacterial changes. We thus tested a clinical strain (529L), isolated from a patient with oral candidiasis [24], which has been previously reported to colonize the oral cavity of healthy mice for over 5 weeks [25]. As seen in Fig 3A strain 529L colonized the tongues of immunocompetent mice at levels similar to strain SC5314 in mice receiving chemotherapy. In healthy mice this strain was found mainly in the superficial epithelial layers (Fig 3C, upper panel) and did not form a visible white biofilm (not shown). We also noticed that this strain formed mostly pseudohyphae under hyphal-inducing conditions *in vitro* (i.e. RPMI, 10%FBS). Despite colonizing the mucosa of healthy mice, this strain was not associated with an increase in viable bacterial counts (Fig 3A) and caused a lower increase in the enterococcal biomass above the untreated control, compared to other experimental groups (Fig 3B). When combined with 5Fu, strain 529L was more invasive (Fig 3C, lower panel) and was associated with a significantly higher increase in enterococcal biomass compared to 529L alone, or 5Fu alone groups (Fig 3B). Enterococci were also detected in sites of tissue invasion with this organism (Fig 3C, lower panel). Although invasive infection was detected histologically, the majority of these mice did not form a visible biofilm lesion (Fig 3C and 3D). These observations indicated that the increased virulence of this strain under conditions of 5Fu-induced immunosuppression and mucosal injury is associated with enterococcal changes.

To confirm these findings we next tested the *tup1Δ/Δ* mutant which forms pseudohyphae and is avirulent in intestinal candidiasis models [17]. Importantly, this mutant acquires virulence in the GI tract with increased dissemination and mortality in a mouse model of immunosuppression combined with intestinal damage [17]. Similar to strain 529L, this strain colonized the tongues of healthy mice (Figs 3A and 1C, upper right panel), and did not cause an increase in the viable bacterial biomass, but was associated with an enterococcal increase similar to 5Fu treatment alone (Fig 3A). Consistent with the GI tract phenotype this strain formed tissue-invasive biofilms in 5Fu-treated mice [17]. Similar to other strains, this mutant co-localized with endogenous enterococci on tongue biofilms (Fig 3C, lower right panel), which were visible as white plaques in some, but not all, mice (Fig 3D). Consistent with the ability to cause invasive biofilms in 5Fu-treated mice, this strain was able to induce an increase in viable bacterial counts (Fig 3A) and a further enterococcal biomass increase, comparable to strain SC5314 under the same conditions (Figs 2E and 3B). In summary these results show that *C*. *albicans* virulence in a host permissive environment is associated with further enterococcal increases in the oral mucosa.

**Fig 4:**
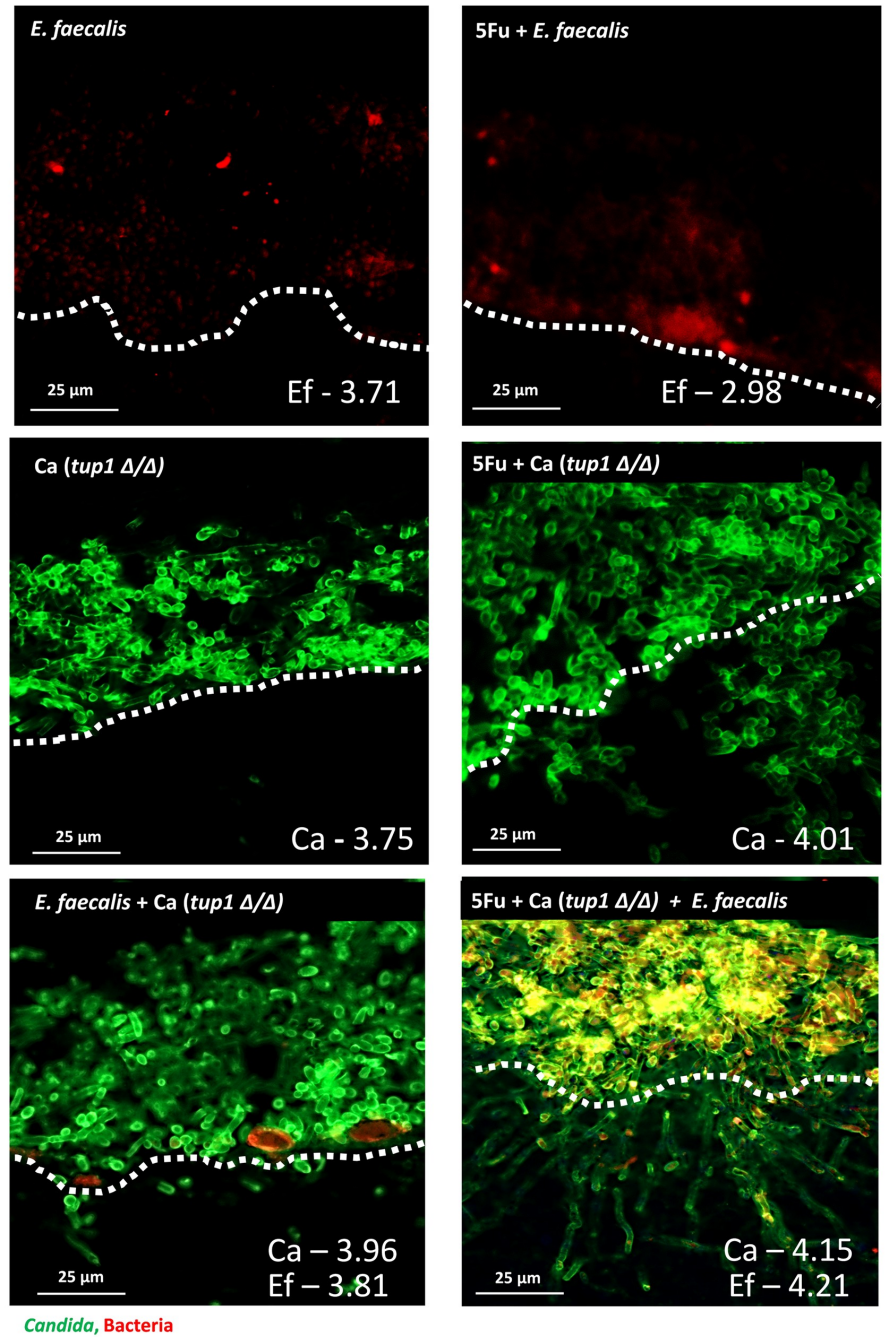
Role of mucosal injury in biofilm growth and invasion using an organotypic model. An *E*. *faecalis* isolate was inoculated alone or in combination with *C*. *albicans* strain *tup1Δ/Δ*. Biofilms are shown growing on mucosal constructs that had been pretreated with 10 μM 5Fu for 16 h (right panel) or on untreated controls (left panel). Tissues were split in half with one half processed for CFU counts and one half for histologic processing and staining. For CFU counts superficially growing biofilms were rinsed off and tissues were weighed, homogenized, and plated. Log_10_ CFU counts corresponding to each tissue and each organism are shown on the lower right. Unwashed tissues were stained with immuno-FISH to simultaneously visualize *C*. *albicans* and *Enterococcus*. *C*. *albicans* was stained with a polyclonal anti-*Candida* antibody (green), and *E*. *faecalis* was stained with EUB338-Alexa 546 probe (red), and areas of co-localization show in yellow. Fungal invasion was more pronounced in 5Fu-treated tissues infected with both organisms and corresponded to bright yellow areas of bacterial and fungal co-localization right above the mucosal breach point. Dotted lines demarcate the start of the submucosal compartment. One of three representative tissues is shown/condition.

Since the 5Fu-induced host permissive environment for *C*. *albicans* entails both mucosal injury and neutropenia we next examined their relative contributions. We first asked whether mucosal injury alone promotes bacterial, fungal or mixed biofilm growth and invasion, using our published organotypic model of 5Fu-induced mucosal toxicity [26]. In this model, oral mucosal constructs were infected with the *tup1Δ/Δ* mutant and an *E*. *faecalis* isolate from mice with 5Fu-associated OPC. As seen in Fig 4, pre-treatment with 5Fu did not significantly affect enterococcal or fungal biofilm growth on the mucosal surface, arguing against a direct role of mucosal injury in promoting biofilms, similar to observations with other bacterial species in this model [26]. However, 5Fu pre-treatment increased invasion of the *tup1Δ/Δ* mutant (Fig 4, right panel). Growth of this strain with *E*. *faecalis* in biofilms promoted invasion into the submucosal compartment of untreated and 5Fu-treated tissues.

**Fig 5:**
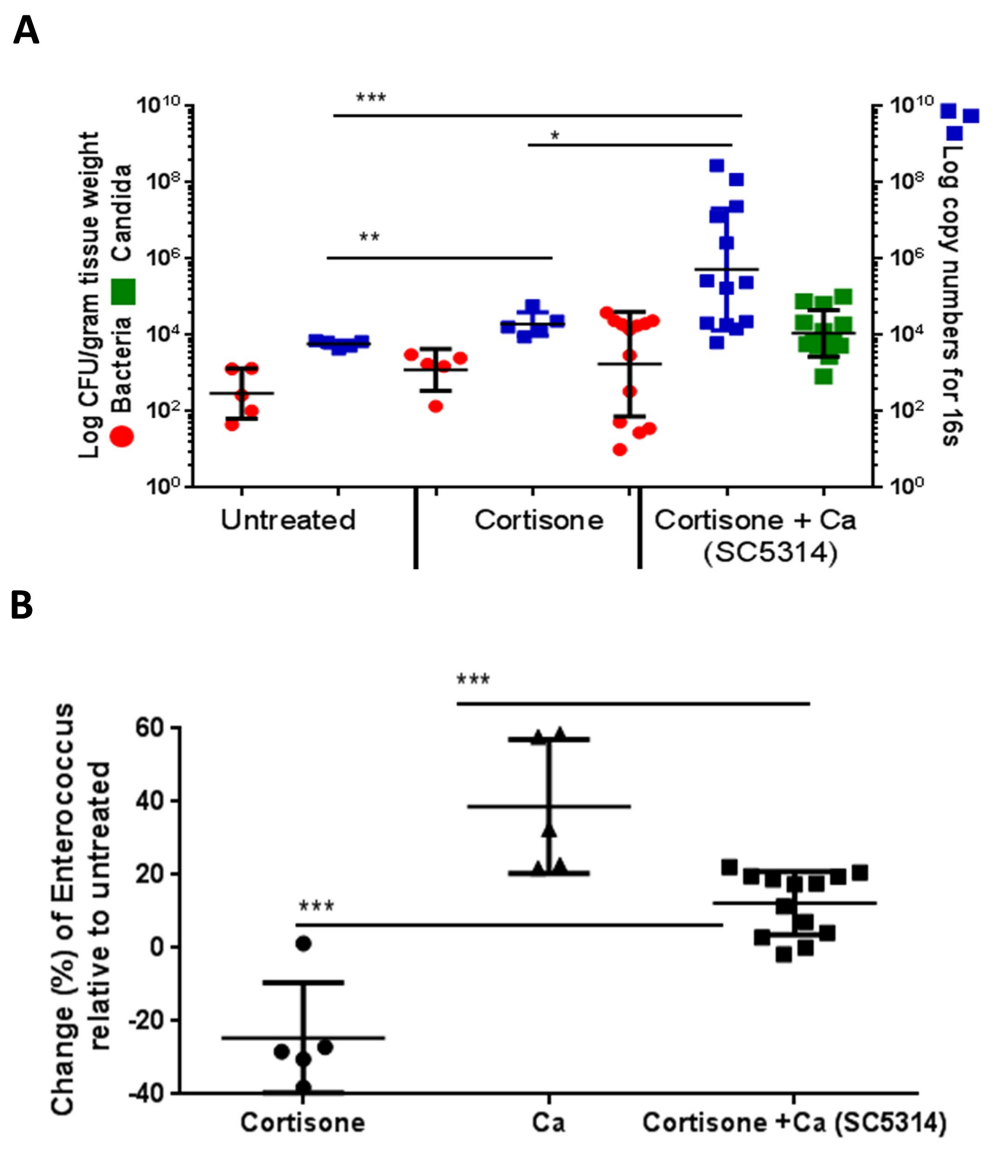
Effect of cortisone immunosuppression and *C*. *albicans* infection on oral bacteria. Mice were immunosuppressed by subcutaneous injection with cortisone acetate and inoculated with *C*. *albicans* SC5314 as described in methods. A: Tongue mucosa-associated bacterial loads in untreated, cortisone-treated, and cortisone-treated *C*. *albicans*-infected mice. Tongues were weighed, homogenized, serially diluted and plated for *Candida* and bacterial burdens. Fungal burdens in infected mice are expressed as log CFU/gm of tissue (green squares, left Y axis). Results for bacteria are expressed as CFU/gm of tissue (left Y-axis, red dots) and log 16S rRNA gene copy numbers/gm of tissue (right Y-axis, blue squares). Bacterial biomass increased with cortisone treatment, but a further significant increase was noted in the cortisone treated + *C*. *albicans* group. Data shown are from 2 independent mouse experiments, with 5–10 mice per group; bars represent means ± SEM. *p<0.05, **p<0.005, ***p<0.002. B: Genus level quantification of *Enterococcus* on tongue tissues by qPCR. Data represent change in percentage of *Enterococcus* load in all groups over untreated control mice. Mice receiving cortisone had a reduction in *Enterococcus* burdens while *C*. *albicans* in cortisone-treated mice raised the burdens close to untreated controls. Results are shown from 5–13 mice per group; bars represent means ± SEM. *p<0.001.

Finally we asked whether immunosuppression alone, in the absence of mucosal injury, was sufficient to induce bacterial biomass and enterococcal changes on the oral mucosa. For this we used the cortisone model, which is not associated with mucosal injury [27,9]. Cortisone treatment alone caused a small but statistically significant increase in the total bacterial biomass on mouse tongues (Fig 5A). Although the total bacterial biomass increased, the enterococcal biomass decreased up to 40% compared to untreated control mice, suggesting that the total biomass increase was due to overgrowth of other bacterial species (Fig 5A and 5B). Importantly, infection with *C*. *albicans* under this type of immunosuppression caused a further increase in total bacterial burdens, while enterococci rebounded at or slightly above untreated levels (Fig 5A and 5B). Taken together these data show that while both types of immunosuppression increase oral bacterial burdens, they influence bacterial biodiversity in different ways. We also conclude that *C*. *albicans* infection favors the growth of enterococci under different immunosuppressive states, regardless of pre-existing mucosal injury.

### *Enterococcus* depletion by antibiotic treatment significantly reduced *C*. *albicans* oral mucosal invasion, without affecting fungal burdens

Since enterococci comprised the vast majority of the bacteria in the tongue mucosa of mice with candidiasis, co-localized with *C*. *albicans* in tissue invasive biofilms, and promoted invasion of the *tup1Δ/Δ* strain in the organotypic mucosa, we asked whether these bacteria are involved in augmenting fungal virulence. We thus hypothesized that an antibiotics regimen which depletes enterococci from the oral mucosa would attenuate fungal virulence. This regimen drastically reduced bacterial CFUs in all groups (S4A Fig). No bacterial DNA amplicons were obtained from tongues in any antibiotics group and few isolates from the group with OPC receiving antibiotics were not enterococci, as determined by 16S sequencing (S4A and S4B Fig).

**Fig 6:**
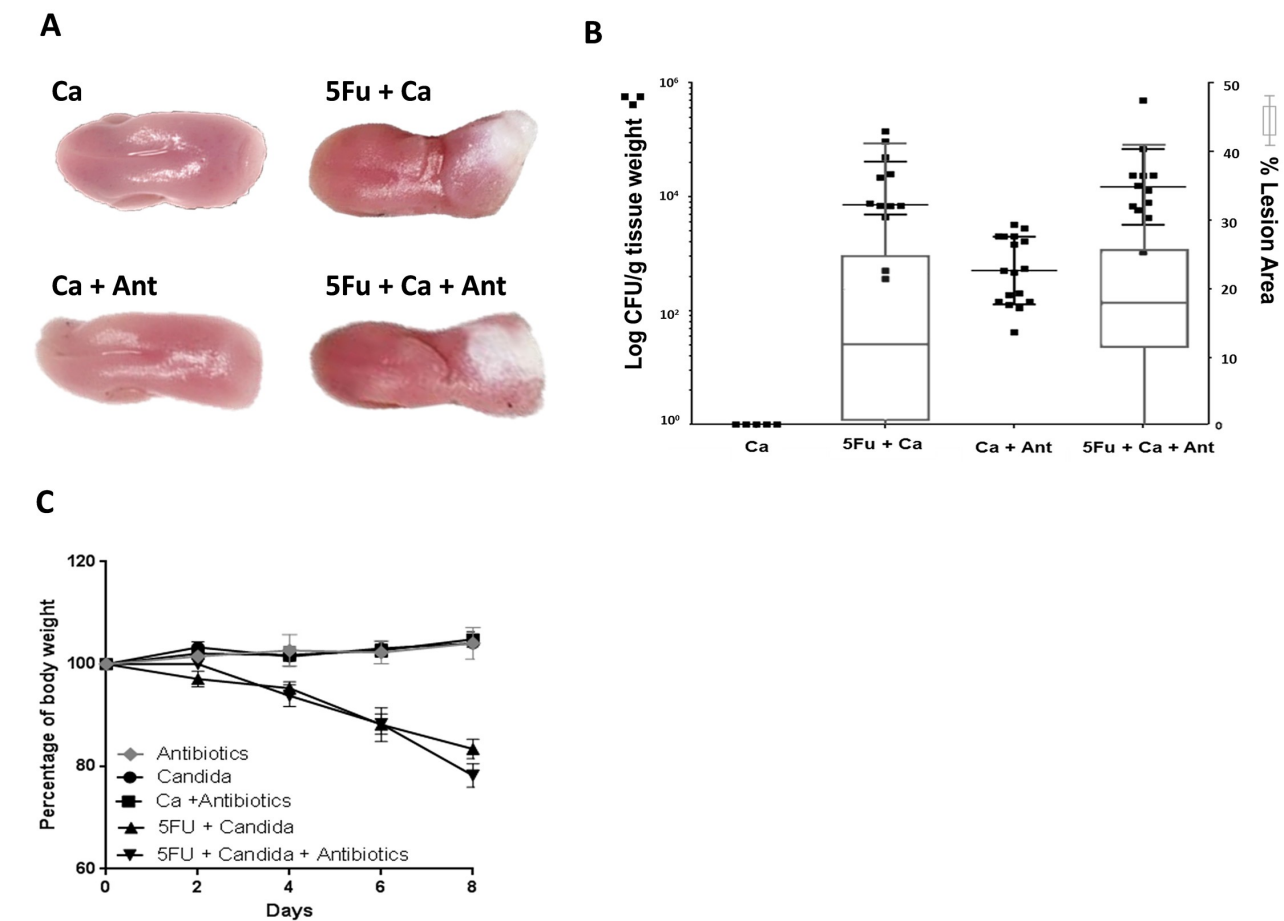
Effect of antibiotic treatment on oral fungal burdens and biofilm surface area in 5Fu-treated mice. Antibiotics were started three days prior to 5Fu-treatment and continued for 8 days. A: Excised tongues after 8 days of *C*. *albicans* SC5314 inoculation. Groups receiving 5Fu with *C*. *albicans* SC5314 (5Fu+Ca) had thick mucosal biofilms covering the posterior tongue surface regardless of antibiotics (Ant) treatment. No biofilms formed in *C*. *albicans*-inoculated healthy mice with (Ca+Ant) or without (Ca) antibiotic treatment. B: Relationship between *C*. *albicans* burdens and biofilm surface area. Figure depicts log fungal CFUs/gm of tissue (dot plot, left Y axis) and corresponding biofilm lesion area (box and whiskers plot, right Y axis) in tongues of four groups of mice (day 8). Mice received *C*. *albicans* SC5314 in the drinking water alone (Ca), 5Fu with *C*. *albicans* (5Fu+Ca), *C*. *albicans* with antibiotics (Ca+Ant) or combination of the three (5Fu+Ca+Ant). Tissues were homogenized, serially diluted and plated for CFU counts. Biofilms were digitally photographed, images were analyzed by Image J and results were expressed as the percentage of dorsal tongue area covered by visible biofilm (white area). In groups treated with 5Fu there were no differences in fungal burdens (p = 0.53) or biofilm area (p = 0.25) between mice receiving antibiotics or not. In mice not receiving 5Fu, antibiotic treatment led to increased *C*. *albicans* burdens (p<0.005) but there was no biofilm lesion on the tongue surface indicating that in healthy mice increased fungal burdens are not sufficient for virulence. Data are from 2 independent mouse experiments, with 5–8 mice per group; bars represent means ± SEM. C: Body weight loss in each group during the eight-day infection period, expressed as percentage of initial weight (day 0) in 5–10 animals per group from 2 independent experiments. Error bars represent SEM. *Candida*-Infected mice receiving 5Fu with or without antibiotics lost 20% of total body weight by day 8.

**Fig 7:**
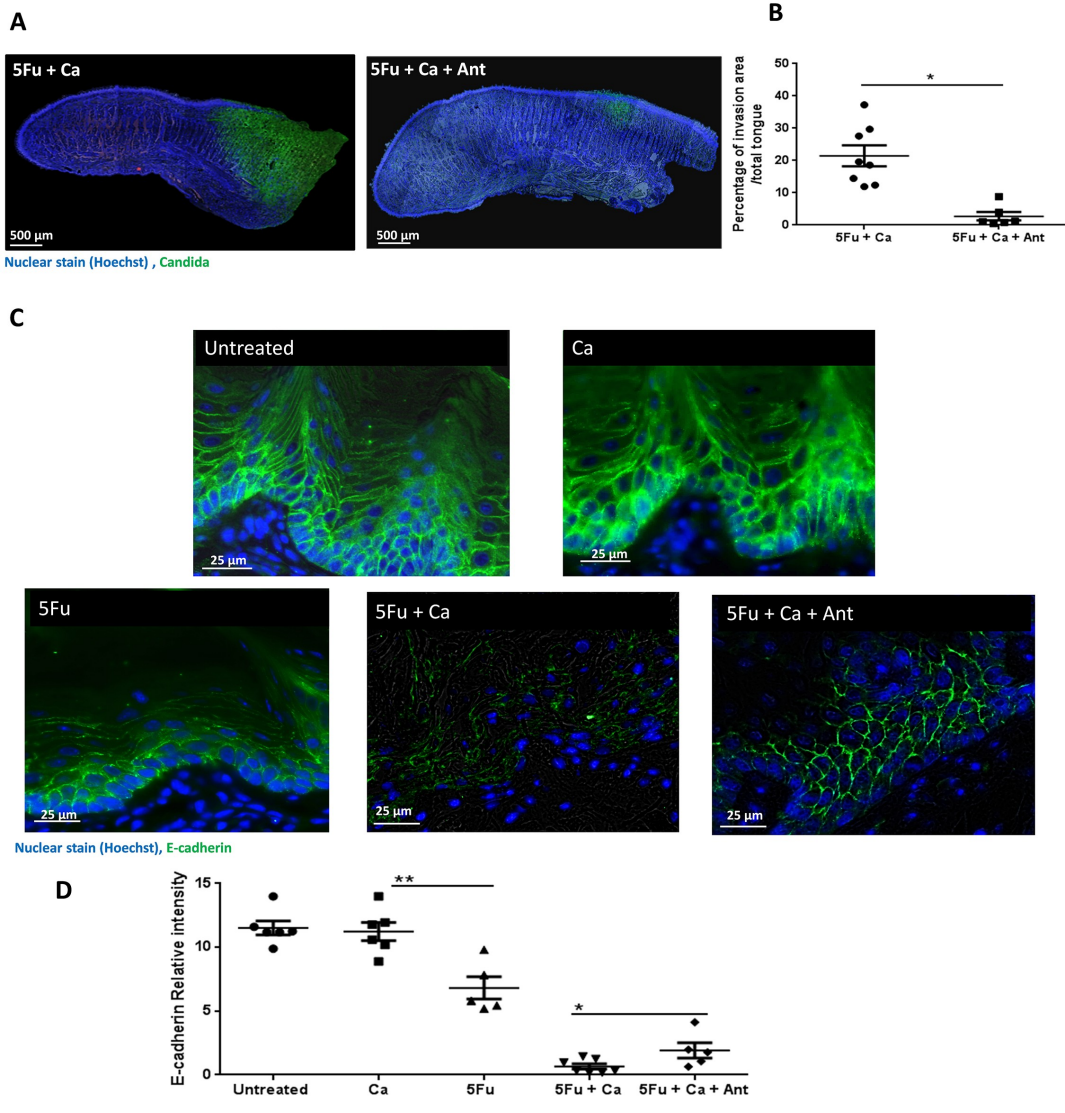
*Enterococcus* depletion by antibiotic treatment significantly reduced oral mucosal *C*. *albicans* invasion and E-cadherin degradation from epithelial adherens junctions. A: Full tongue sagittal section scans from mice receiving 5Fu with *C*. *albicans* SC5314 (5Fu+Ca) or mice additionally receiving antibiotics (5Fu+Ca+Ant) 8 days after initiation of 5Fu and fungal inoculation. Tongues were stained by immunofluorescence with an anti-*Candida*-FITC polyclonal antibody (green), and counterstained with Hoechst 33258 (blue) to visualize cell nuclei. Note the reduction in *C*. *albicans* invasion into the submucosal tissues with antibiotic treatment. B: Tongues stained as above were analyzed by Image J and the percentage of area invaded by fungi (green over blue signal) was calculated. Data is from 2 independent mouse experiments, with 3–5 mice per group; bars represent means ± SD. *p<0.001. C: Tongue tissue sections from untreated mice, mice receiving 5Fu alone, mice inoculated with *C*. *albicans* SC5314 alone (Ca), mice receiving 5Fu and *C*. *albicans* (5Fu+Ca) or mice additionally receiving antibiotics (5Fu+Ca+Ant) sacrificed 8 days after initiation of 5Fu and fungal inoculation. Tongues were stained by immuno-fluorescence for E-cadherin (green) and counterstained with nucleic acid stain Hoechst 33258 (blue). E-cadherin signal was intact in untreated mice and healthy mice inoculated with *C*. *albicans*, indicating integrity of adherens junctions. Signal was reduced in all other groups, with the greatest reduction in mice receiving 5Fu and *C*. *albicans*. D: E-cadherin staining was analyzed by ImageJ and mean intensity of the green signal per mm^2^ was calculated in tissue sections from 6 mice/group. Bars represent means ± SD. *p<0.05, **p<0.005.

Treatment of immunocompetent mice with antibiotics increased *C*. *albicans* burdens on the tongue, as previously observed by others [reviewed in 28], but did not lead to development of white mucosal biofilms or weight loss (Fig 6A, 6B and 6C). In contrast, in 5Fu-treated mice inoculated with *C*. *albicans* antibiotics did not cause changes in fungal loads, or in the biofilm surface area, compared to mice not receiving antibiotics. Even though the biofilm surface area and fungal loads were similar in the two chemotherapy groups tissue invasion of *C*. *albicans* in mice receiving antibiotics was significantly attenuated (Fig 7A and 7B). However, both chemotherapy groups had diarrhea and lost a significant amount of weight, consistent with an absence of a protective effect of antibiotics on the intestinal mucosa [17] (Fig 6C).

Our earlier work demonstrated that oral mucosal invasion of *C*. *albicans* is associated with E-cadherin degradation from epithelial adherens junctions [29] and that one way whereby commensal streptococci can augment tissue invasion is by synergistically activating host enzymatic pathways to degrade this protein [8]. Since antibiotics attenuated the invasive phenotype of *C*. *albicans* on the tongues we reasoned that they would attenuate E-cadherin degradation. As we showed previously in this model [19], 5Fu treatment alone induced a significant loss of the E-cadherin signal in the oral mucosa (Fig 7C and 7D). Invasive infection in 5Fu-treated mice was associated with almost complete E-cadherin dissolution, whereas protein integrity in adherens junctions was partially preserved with antibiotics treatment (Fig 7C and 7D). In summary, these results suggest that the dysbiotic bacterial microbiota present in the oral mucosa of mice with OPC may contribute to the E-cadherin degradation and increased *C*. *albicans* invasion in the 5Fu model.

### *Enterococcus* isolates from dysbiotic mucosal biofilms degrade E-cadherin and facilitate *C*. *albicans* transepithelial invasion

**Fig 8:**
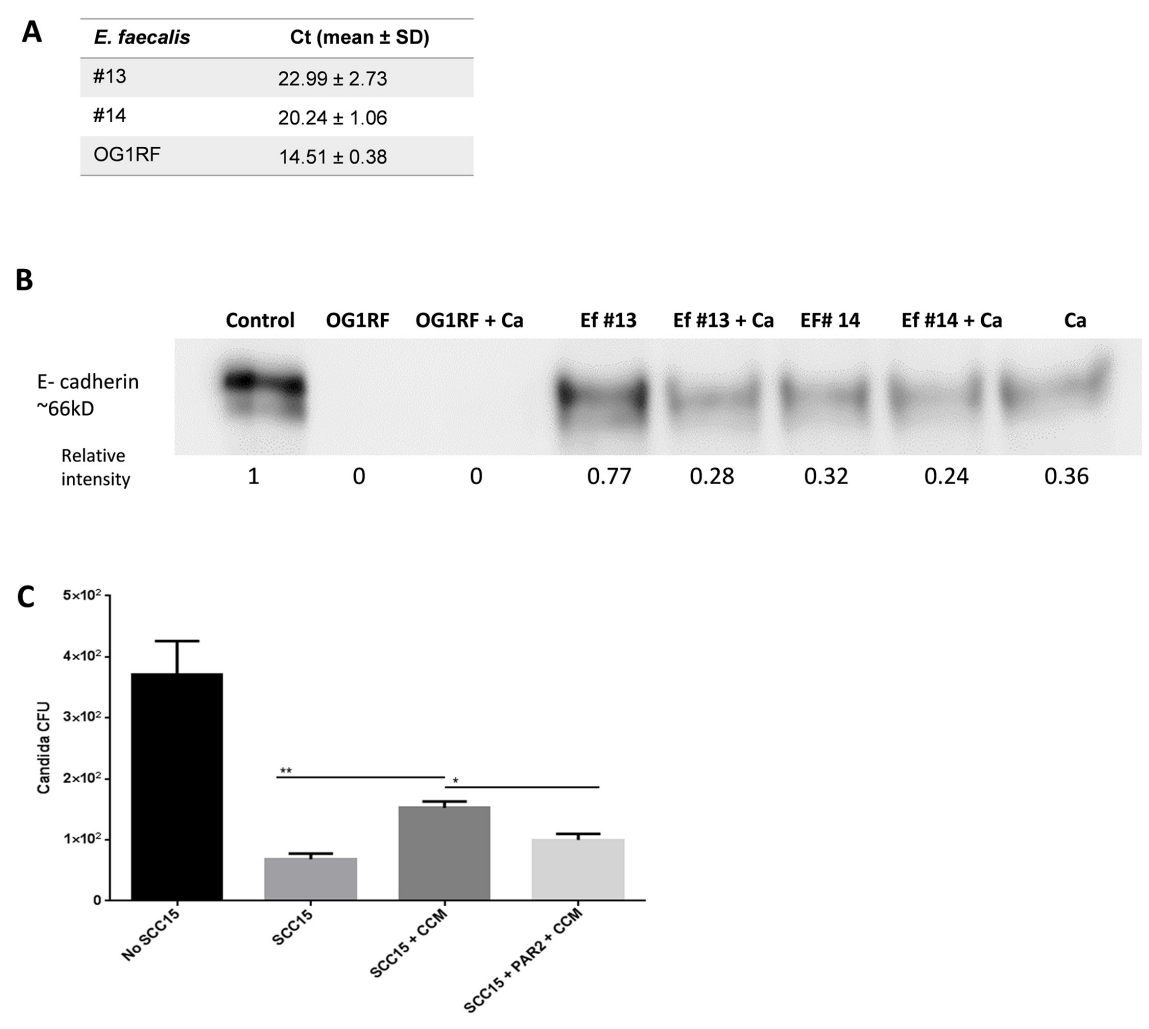
Enterococcus isolates from oral dysbiotic communities are able to degrade E-cadherin and promote epithelial permeability. A: Analysis of *E*. *faecalis* gelE gene expression by quantitative RT-PCR. Comparison of gene expression levels in stationary phase overnight cultures of isolates #13 and #14, with strain OG1RF used as a positive control. Relative RNA levels were calculated using the DDC_t_ method and recA gene expression was used as an internal control to normalize RNA concentration. Data were expressed as mean threshold cycle (C_t_) ± SD of duplicate samples in at least three independent cultures. B: E-cadherin degradation assay. Concentrated conditioned media from stationary phase cultures of *E*. *faecalis* isolates #13 and #14 were incubated with 3 μg of recombinant E-cadherin for 1 hour, with or without conditioned media from *C*. *albicans* SC5314. *E*. *faecalis* strain OG1RF was used as a positive control. Western blot shows reduction of E-cadherin signal with both isolated strains and complete degradation of the protein with strain OG1RF. Numbers under each lane represent relative image density as measured with the Image J software. One representative of three experiments is shown. C: Migration of *C*. *albicans* (SC5314) across confluent SCC15 cell monolayers in a transwell assay. Fungi translocating to the lower chamber after 4 hours incubation were quantified by plating the media for CFUs. *C*. *albicans* migration was significantly greater when monolayers were apically pretreated with *E*. *faecalis* Concentrated Cell Conditioned Media (CCM) compared to untreated or CCM+PAR2 inhibitor pretreated controls. Results are from 2 independent experiments with 3 technical replicates in each. Data are expressed as mean values and SEM. *p<0.05, **p<0.0001.

One of *E*. *faecalis* virulence attributes which contributes to loss of intestinal barrier function, is secretion of the metalloproteinase GelE, involved in extracellular domain E-cadherin degradation [30]. Intestinal epithelial cell permeability by this enzyme was shown to be mediated by via protease activated receptor 2 (PAR2) [31]. Although most *E*. *faecalis* strains have a copy of this gelatinase gene, expression varies significantly from strain to strain [32]. Thus we tested two *E*. *faecalis* isolates (isolates #13 and #14) from chemotherapy-treated mice with OPC for gelE expression under standard growth conditions and asked whether conditioned media from these isolates could degrade recombinant E-cadherin. GelE was assessed at the transcript level and compared to strain OG1RF which expresses very high levels of this enzyme [31,30]. Both oral isolates expressed this gelatinase, albeit at much lower levels than strain OG1RF, consistent with other reports on other *E*. *faecalis* isolates (Fig 8A) [30,32]. In accordance with GelE expression levels, recombinant E-cadherin was degraded by conditioned media from *E*. *faecalis* isolates in variable degrees, whereas strain OG1RF completely degraded this protein. Treatment of recombinant E-cadherin with conditioned media from *E*. *faecalis* isolates and *C*. *albicans* together, further degraded this protein (Fig 8B). In the next series of experiments we asked whether conditioned media of the two organisms alone or in combination could increase the permeability of oral epithelial cells in a transwell monolayer assay. Following pre-treatment of the cells with *E*. *faecalis* conditioned media we first measured the flux of FITC-labeled dextran, added on the upper chamber, across the monolayer. *E*. *faecalis* conditioned media promoted the permeability of the epithelial monolayer. Importantly, a PAR2 antagonist partially but significantly inhibited the permeability induced by *E*. *faecalis*, suggesting a role for gelatinase E in this process (S5 Fig). Finally, we found that *C*. *albicans* translocation across the epithelial layer was greater in cells pre-treated with *E*. *faecalis* conditioned media and that this was attenuated by the PAR2 antagonist (Fig 8C). Taken together these results support the hypothesis that *E*. *faecalis* at least partially contributes to mucosal barrier breach.

## Discussion

**Fig 9:**
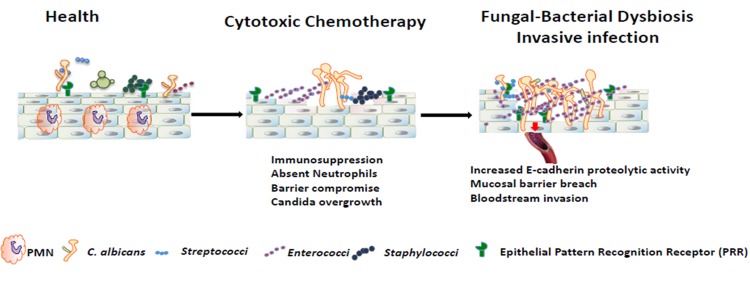
Model of oral mucosal candidiasis in chemotherapy. Cytotoxic chemotherapy promotes a dysbiotic state characterized by overgrowth of *C*. *albicans* and certain resident bacterial species. Bacterial proteolytic activity promotes mucosal barrier breach by *C*. *albicans*.

In this study we investigated the influence of *C*. *albicans* infection on the composition of the oral and intestinal mucosa-associated bacteria in the context of cytotoxic chemotherapy. We demonstrated that *C*. *albicans* infection led to a profound taxonomic imbalance on the oral mucosa that contributed to pathology. We also discovered that antibiotics that clear the dominant *Enterococcus* taxon during infection ameliorate invasive candidiasis, further demonstrating that bacterial community changes in OPC represent a dysbiotic shift promoting *C*. *albicans* virulence. Importantly, although antibiotics alone significantly increased oral fungal burdens, virulence required a chemotherapy-modulated host environment. Mucosal injury and immunosuppression caused by 5Fu played a major role in increased invasive infection in this model, with dysbiotic communities playing an accessory role. Thus our studies support a novel pathogenesis framework in the oral mucosa which includes the fungus, the resident bacterial microbiota and a host-permissive environment (Fig 9).

One of the limitations of the 5Fu model is that bone marrow and mucosal toxicity are simultaneously occurring in the same host such that it is extremely difficult to dissect their independent contributions in dysbiosis. A comparison of the 5Fu and cortisone models showed that both types of immunosuppression allow overgrowth of endogenous bacterial organisms on the oral mucosa. However the two treatments had different effects on enterococci, with 5Fu promoting and cortisone curtailing their growth, supporting qualitatively different effects on biodiversity. This is not surprising since mice given cortisone have an increased number of functionally competent infiltrating neutrophils, whereas mice given cytotoxic chemotherapy are severely neutropenic [19, 9, 33, 34]. Given the central role of neutrophils in the control of endogenous enterococcal overgrowth in mice [35], the finding that a neutropenic state induced by 5Fu led to overgrowth of these organisms was anticipated. Alternatively, mucosal injury could be involved in the differences observed between the two types of immunosuppression. Although studies in the organotypic model did not support this alternative, the model lacks vascular and immune components and cannot fully recapitulate in vivo conditions. On the other hand, *C*. *albicans* infection increased growth of enterococci in both immunosuppressive states, suggesting a mutualistic relationship of the two organisms, independent of immunosuppression. This was further supported by the fact that healthy mice inoculated with *C*. *albicans* daily via the drinking water had an increase in the endogenous enterococcal biomass on the oral mucosa. We conclude that both immunosuppression and *Candida* play a direct role in bacterial changes but increased fungal burdens due to 5Fu treatment further amplify bacterial biomass changes.

This is one of the few studies that compared the effects of *C*. *albicans* inoculation on oral and intestinal sites of the same mice, and the only study to perform comparative global microbiome analyses in health and immunosuppression. We found that in healthy mice inoculated with *C*. *albicans* bacterial community diversity decreased in the oral and increased in the small intestinal mucosa. This may reflect differences in the resident bacterial composition prior to inoculation, or differences in the interaction of *C*. *albicans* with distinct innate mucosal host environments. In contrast, in a disease-permissive host environment the most important driver of community changes was *C*. *albicans* since infection led to reduction in bacterial diversity and enterococcal expansion both in the oral and intestinal mucosa.

A previous study of changes in the intestinal microbiota in response to 5Fu in mice used a single high dose intraperitoneal injection and analyzed stool and not intestinal mucosa-associated bacteria [36], thus our analyses are not directly comparable. In our model emphasis was placed on mucosa-associated bacteria with the potential to form mucosal biofilms with *C*. *albicans*. Our focus on jejunum instead of other parts of the intestinal mucosa was due to the relevance of this site to mouse intestinal mucositis models [37]. Our taxonomic profiles showed a dominance of *Enterococcus* on the oral mucosa, with all of our isolates being *E*. *faecalis*. Like *C*. *albicans*, *Enterococcus* species are a major concern in critical care areas due to resistance to multiple antibiotics [38,39,40]. In the human oral cavity enterococci are generally considered transient commensals and carriage rates in healthy adults are below 10%. However, the oral carriage rate of *Enterococcus* species (predominantly *E*. *faecalis*) in patients with underlying systemic disease such as diabetes or cancer rises up to 60% [41,42,43,44,45]. These are also some of the most high-risk populations for OPC [reviewed in 46]. In fact, there is mounting evidence that *C*. *albicans* and *Enterococci* co-exist in human disease samples [reviewed in 47]. A large-scale retrospective analysis showed that *E*. *faecalis* was twice as likely to be isolated in *Candida*-positive sputum and even more likely in sepsis [48]. Other studies have shown *E*. *faecalis* and *C*. *albicans* co-isolated in >10% of root canal infections [49] and in >40% of human tongue mucosal lesions [50]. Whether, in addition to pathogenic synergy, this co-isolation reflects similar host adaptation strategies remains unclear.

The only published model of cytotoxic chemotherapy that assessed oral and gastrointestinal candidiasis in the same mice included wide spectrum antibiotics, which precluded assessment of the role of candidiasis on bacterial communities or the role of bacteria in disease progression [51]. One of the known consequences of mucosal injury and neutropenia in chemotherapy models is systemic dissemination of *C*. *albicans* to distant organs through the gastrointestinal mucosa [17,18]. However, studies that examined this effect of chemotherapeutic agents also used antibiotics which by themselves reduce gastrointestinal mucosal barrier function [52,53]. Our model thus offered the opportunity to examine the effects of chemotherapy on *Candida* dissemination in mice with unperturbed commensal microbiota.

As one regulator of mucosal barrier function E-cadherin is targeted by both mammalian and microbial metalloproteases [54]. In proof of principle experiments we tested the proteolytic activity of our isolates and showed their ability to partially degrade E-cadherin *in vitro*. Compromised epithelial junction integrity mediated by GelE activity is a key step in *E*. *faecalis* intestinal pathogenesis [30,55,56]. Despite the epithelial junction integrity consequences, these studies observed no substantial penetration of *E*. *faecalis* in the inner mucus layer of the intestinal mucosa, consistent the lack of oral mucosal invasion by enterococci in our model. Oral isolates from mice with OPC showed low levels of gelE expression, suggesting this enzyme does not play a major role in breaching the oral mucosal barrier. Activation of host metalloproteinases by dysbiotic bacteria may represent an alternative mechanism of synergistic mucosal barrier destruction [57]. In addition, high amounts of reactive oxygen metabolites produced by enterococci can amplify epithelial damage [58]. In intestinal pathogenesis models *E*. *faecalis* has also been shown to have proinflammatory consequences by signaling through TLR2 to induce IL-6 [59,60,61] or by increasing expression of TLR4, which in theory would increase proinflammatory signals by Gram (-) bacteria [62] These proinflammatory attributes of *E*. *faecalis* may contribute to the increased proinflammatory cytokine production in the tongues of mice with OPC.

In conclusion we identified distinct bacterial microbiota profiles associated with *C*. *albicans* infection in the context of cytotoxic chemotherapy. We also discovered that changes in the resident bacterial microbiota contribute to OPC pathogenesis, thus representing a dysbiotic state. This is the only study to experimentally and systematically dissect the role of *C*. *albicans* in shaping resident bacterial communities in an immunosuppressed host and the reciprocal role of mucosal bacteria in fungal pathogenesis.

## Materials and methods

### Ethics statement

All animal studies were performed in compliance with the federal regulations as described in the Animal Welfare Act (AWA), the recommendations in the Guide for the Care and Use of Laboratory Animals of the National Institutes of Health, and the guidelines of University of Connecticut Institutional Animal Use and Care Committee (IACUC). All protocols used in this study were approved by the IACUC committee of UCONN Health, IACUC protocol #101640–0720.

### Microorganisms and microbiological media

*C*. *albicans* SC5314 (kindly provided by Dr. Aaron Mitchell), is a laboratory strain originally isolated from a patient with bloodstream infection [63]. *C*. *albicans* strain 529L, is a strain originally isolated from a patient with oral candidiasis (kindly provided by Dr. Marc Swidergall, UCLA). *C*. *albicans tup1Δ/Δ* homozygous deletion mutant is from the SC5314 parental background (kindly provided by Dr. Alexander Johnson, UCSF). *Candida* strains were routinely maintained in yeast extract-peptone-dextrose (YPD, BD Difco) agar and overnight stationary phase cultures were prepared in YPD broth prior to each experiment [64]. *E*. *faecalis* OG1RF (kindly provided by Dr. Margaret Vickerman, SUNY Buffalo), and mouse bacterial isolates were cultivated to early log phase in brain heart infusion medium (BHI, BD Difco) at 37°C under aerobic conditions, prior to each experiment.

### Mouse model

We used an intravenous chemotherapy mouse model that recapitulates mucosal and bone marrow changes in cancer patients receiving 5-fluorouracil [19]. In this model, we tested the ability of *C*. *albicans* supplied in the drinking water to cause oral, gastrointestinal and disseminated candidiasis (animal protocol 101640–0720). Six to nine-week-old female C57BL/6 mice (Jackson Labs) were used (animal protocol 101640–0720). Mice received 50 mg/kg 5Fu (Sigma), intravenously (IV, via lateral tail vein) every 48 hours, for 8 days. This mode of administration was previously optimized to induce oral and small intestinal mucositis recapitulating cancer chemotherapy-associated mucositis in humans [19, 65]. Control groups received PBS IV. Mice were inoculated with an overnight *C*. *albicans* suspension culture added daily in the drinking water (6 × 10^6^ yeast/ml of water). Chemotherapy-naive mice were inoculated with *C*. *albicans* strain 529L according to a sublingual protocol that allows stable colonization in a commensal state [25] and a suspension culture was also added daily in the drinking water. In some experiments 5Fu-treated mice received a combination of three antibiotics (Penicillin 1.5 mg/mL, Streptomycin 2 mg/mL and Gentamicin 0.1 mg/ml) in their drinking water, starting three days prior to fungal inoculation and continuing throughout the experimental period.

For cortisone immunosuppression mice received two subcutaneous injections with cortisone acetate (225 mg kg^−1^) on the first and third day of the experiment. On the second day mice were anaesthetized by an intramuscular injection of ketamine: xylazine (90–100 and 10 mg kg^−1^ of body weight, respectively) and a small cotton pad soaked with 100 μl of a *C*. *albicans* SC5314 cell suspension (6 × 10^8^ yeast ml^−1^) was placed under the tongue for 2 hours. During the following 5 days animals were also given drinking water containing a daily-fresh suspension of *C*. *albicans* as above. Organs were retrieved after sacrifice at each time point. Experiments were repeated at least twice with a minimum of 4 mice per group.

### Quantification of mucosa-associated *C*. *albicans* and bacteria

#### Cultivable microbial quantification

Tongues, esophagi and jejunum were rinsed twice with sterile PBS, to discard loosely adherent microorganisms. For fungal CFU quantification homogenized tissue aliquots were plated on Sabouraud Dextrose Agar (SDA) containing chloramphenicol (10 μg/mL, Sigma). In some experiments homogenates were also plated on CHROMagar medium (DRG International) to rule out overgrowth of endogenous non-albicans *Candida* species. Cultivable bacterial counts were performed using aliquots of the same samples on BHI Agar supplemented with Nystatin (250 U/mL, BHYN) [66]. Bacterial cultures were incubated anaerobically for 5 days [67].

#### Bacterial qPCR

Bacterial genomic DNA from mouse tissues was prepared by using an overnight lysis protocol optimized for oral mucosal microbiome characterization in murine models [68]. Tissues were further processed using the Qiagen DNA Blood and Tissue mini kit. DNA quantity and quality were evaluated using a NanoDrop. Total bacterial biomass (16S rRNA gene copy numbers) was quantified by qPCR with primers, TaqMan probe sequence and thermal cycling procedures as previously published [69]. For enterococcal biomass quantification we performed qPCR using the genus-specific primers ECST748F AGAAATTCCAAACGAACTTG and ENC854R CAGTGCTCTACCTCCATCATT and probe GPL813TQ 6FAM-TGGTTCTCTCCGAAATAGCTTTAGGGCTA-TAMRA as previously described [70]. All reactions were performed in duplicate and standards were prepared using serially diluted genomic DNA from *E*. *faecalis* strain OG1RF.

### Bacterial microbiome evaluation

DNA extracted from tissues was quantified using the Quant-iT PicoGreen kit (Invitrogen). 16S rRNA genes were amplified in triplicate using 30ng of extracted DNA as template. The V4 region was amplified using 515F and 806R primers with Illumina adapters and bar codes on the 3’ end [71]. PCR products were pooled for quantification and visualization using the QIAxcel DNA Fast Analysis kit (Qiagen). Pooled PCR products were processed using the Mag-Bind RxnPure Plus kit (Omega Bio-tek) according to the manufacturer’s protocol, to include only sequences between 250–400 bp. The cleaned pool was sequenced on the MiSeq using v2 2x250 base pair kit (Illumina).

### Sequence analysis

Sequences were processed following a standard pipeline [72] and classified using Mothur’s version of the Ribosomal Database Project classifier (Mothur 1.39.5) [73]. For operational taxonomic unit (OTU) analyses, sequences were clustered using a 97% similarity cutoff and classified up to genus level based on the consensus taxonomy. Alpha and beta diversity statistics were calculated by subsampling 1000 reads per sample. Alpha-diversity was measured via Shannon diversity index, and community richness as average OTUs (species). The relative abundance of OTUs of main genera was determined to assess the effect of treatments. NMS was used to observe community clusters associated with Bray-Curtis dissimilarity calculations in different tissues of the same mice undergoing different treatments. Association between Shannon diversity index and bacterial CFUs was tested using linear regression. NMS plots were used to survey bacterial OTU heterogeneity relative to each treatment at each time point and permutational ANOVA (PERMANOVA) comparisons of the Bray Curtis dissimilarity distances were performed. Community structures across groups were visualized in standard graphing packages within R, version 3.2 (http://www.r-project.org).

### Isolation and Identification of cultivable bacteria

Isolates were selected on the basis of colony and cell morphology after Gram staining, and stored in 20% glycerol at -80°C. Isolates were identified at the genus level by sequencing, as described above. Species level identification was performed for *Enterococcus* isolates by PCR using species-specific primers as described elsewhere [74].

### Histological staining

Organisms were visualized by immunofluorescence staining combined with FISH, as we describe in detail elsewhere [75]. Briefly, tissues were stained with a FITC-labeled anti-*Candida* polyclonal antibody (Meridian Life Science), the oligonucleotide probe EUB338 labeled with Alexa 546, targeting all bacteria [76], the *Enterococcus/Lactobacillus* probe LAB158 labeled with Alexa 546, and the *E*. *faecalis* probe ENFL84 labeled with Alexa 405 [77]. To evaluate the integrity of the mucosal barrier, E-cadherin was assessed by immunofluorescence staining using a polyclonal antibody (BD Biosciences) followed by a fluorescein isothiocyanate-conjugated secondary antibody (DyLight 488; Vector Laboratories, Burlingame, CA) as described previously [19]. Neutrophils were visualized in fresh frozen sections with a monoclonal antibody (NIMP-R14, Hycult), followed by a secondary antibody conjugated with Alexa 555 (A-21434, Invitrogen). Tissues were counter-stained with the nucleic acid stain Hoechst 33258 (Invitrogen). Images were obtained using a Zeiss Axio Imager M1 microscope and an EC-Plan-Neofluar 920-NA 0.5 air-objective. ImageJ software was used to quantify signal intensity in a minimum of 5 sections/condition.

### Organotypic mucosa infection model

Oral mucosal organotypic constructs that mimic non-keratinized stratified human oral mucosa have been described elsewhere [78]. Briefly, the constructs consist of SCC15 oral keratinocytes (American Tissue Culture Collection, ATCC) seeded on collagen type I–embedded fibroblasts (3T3, ATCC). Tissues are airlifted for 2–3 weeks prior to infection. Tissues were inoculated with 10^6^ cells of *C*. *albicans* SC5314, 10^7^ cells of bacteria, or a combination, and incubated for 20h. Pre-treatment of some tissues with 10 μM 5Fu overnight was performed prior to microbial inoculation as detailed elsewhere [26]. Prior to inoculation 5Fu was removed by washing tissues three times in PBS. For CFU determinations of invading organisms, superficially growing biofilms were removed by gentle washing with sterile PBS prior to tissue weighing, followed by homogenizing and plating.

### E-cadherin degradation assay

*E*. *faecalis* isolates from 5Fu-treated mice with candidiasis (#13, and #14) and *E*. *faecalis* strain OG1RF (positive control) [30] were grown overnight to stationary phase in BHI broth. Concentrated culture-conditioned media (CCM) were concentrated 25 times and 50μl of each were incubated with purified recombinant human E-cadherin (3μg) (Sigma) at 37°C for 1h. As control, E-cadherin protein was incubated with BHI broth (50μl). Protein degradation was detected by Western blot using E-cadherin intercellular junction marker antibody (ab40772, Abcam) and goat anti-rabbit IgG-HRP (ab6721, Abcam).

### gelE RT-qPCR

*E*. *faecalis* cultures were grown overnight in BHI broth to OD = 1. Bacterial pellets were harvested by centrifugation and washed with sterile PBS. RNA was isolated with the RNeasy mini kit (Qiagen) followed by DNAse digestion using the Turbo DNA free kit (Invitrogen). Equal amounts of RNA were converted to cDNA using Superscript III first strand synthesis kit (Invitrogen). Q-PCR was performed using CFX96- Real time system (Biorad) and data were normalized using recA as internal control. The primers used were as follows: recA: FP- GCAACGAAATGGTGGAACAG, RP- AAGGCATCGGCAATCTCTAAG [79], gelE: FP- CGGAACATACTGCCGGTTTAGA, RP- TGGATTAGATGCACCCGAAAT [80].

### Epithelial permeability assay

SCC15 cell monolayers were seeded at 5 x10^4^ cells/well in 0.2 ml of KSFM media on the upper chamber of the transwell (Millicell 0.45 μm pore size, Millipore), and 0.5 ml of media were added in the bottom of the well. Epithelial cells were allowed to adhere and form a monolayer at 37°C in 5% CO_2_. Next day, cells were exposed to PAR2 antagonist (FSLLRY-NH2; Tocris, Bristol, United Kingdom) for 24 hours, at a final concentration of 20 μM [31]. Concentrated conditioned media from *E*. *faecalis* isolates were prepared as described elsewhere [31] and added apically for 16 hours. Permeability was measured by fluorescein isothiocyanate (FITC)-labeled dextran (78 × 10^3^ ng/ml) added to the upper chamber, and monolayers were incubated for 4 hours. Fluorescence was measured in the lower chambers of the transwell plates using a fluorometer (Synergy 2, by BioTek with Gen5 Software) at an excitation wavelength of 485 nm and an emission wavelength of 530 nm to determine the flux of FITC-dextran across the monolayer, based on a previously prepared standard curve.

*Candida albicans* transmigration assay through the monolayer was performed under the same conditions, with a 5.0 μm pore size transwell insert (Costar, Corning 3421). Following treatments with PAR2 inhibitor and conditioned media from *E*. *faecalis* as above, transwells received 10^4^ cells/well of *C*. *albicans* which were allowed to translocate to the lower chamber for 4 hours. *C*. *albicans* migration through the SCC15 monolayer was quantified by CFU counts from the lower chamber of the transwells.

### Statistical analyses

Statistical significance was determined by two-tailed t-test, assuming equal variances, or the Mann Whitney test when data were not normally distributed.

### Supplemental figure methods

#### Flow cytometry

For flow cytometry staining blood was collected by retro-orbital bleeding. Tongue and bone marrow cells were obtained after sacrifice at the indicated time points. Bone marrow was flushed with PBS and tongue tissues were disrupted using a tissue homogenizer in PBS. Red blood cells were lysed by adding lysis buffer (8.26 g NH4Cl, 1.19g NaHCO3 in 100 mL of water for a 10x solution) for 10 min. Supernatant was discarded and pellet was washed in FACS buffer composed of phosphate-buffered saline (PBS) (Gibco), 5% FBS, and 0.5% sodium azide, for 20 min at 4°C. Fc Receptors were blocked before staining with the anti CD16/32 (Clone 2.4G2) antibody in FACS buffer. Pellets were resuspended in FACS buffer with a combination of the following antibodies: CD45 -PerCPCy5.5, Ly-6G-FITC, CD11b-APC (all from Biolegend). Finally, Live/dead cells were stained with Zombie violet for 20 min at 4°C, samples were fixed for 5 min at 4°C with 2% PFA in PBS and kept in 4°C until sorting. Data were analyzed by FlowJo software.

#### Multiplex cytokine ELISA

Tissues were homogenized in Mammalian Protein Extraction Reagent (Thermo Fisher Scientific) and a protease inhibitor cocktail (cOmplete tablets, Sigma-Aldrich), with a POLYTRON homogenizer, followed by 10 minutes disruption in a bead beater and sonication for 1 min. Samples were centrifuged at 13000 rpm for 5 min, and total protein concentration was quantified using the BCA protein assay kit (Thermo Fisher Scientific). Samples were diluted as needed in order to standardize the amount of protein. IL-6, keratinocyte chemoattractant (KC) and Tumor Necrosis Factor-alpha (TNFα) were simultaneously quantified in each sample using the Luminex/MAGPIX system (RCYTOMAG-80K; Millipore).

## Supporting information

S1 Fig Effect of 5Fu administration and *Candida albicans* (SC5314) infection on neutrophil counts and chemokine expression. A: Time-dependent analysis of tongue neutrophils. Mice receiving 5Fu (50mg/kg, IV, every 48 hours) + *C*. *albicans* (5Fu+Ca), and either 5Fu or *Candida* alone were sacrificed at the indicated time points and CD11b+/Ly6G+ cells extracted from tongues were sorted by FACS. Note the almost complete depletion of tongue neutrophils in the 5Fu+Ca group by day 6. B: Time-dependent analysis of neutrophil numbers in tongue, bone marrow and peripheral blood. In tongue tissues the 5Fu and *Candida* alone groups had a steady influx of neutrophils, slightly higher than the PBS control on days 2 and 6, whereas a dramatic drop was noted in the 5Fu+*Candida* group by day 6. In the bone marrow mature cells declined rapidly in both groups receiving 5Fu, consistent with the myelosuppressive action of this drug. Blood counts paralleled those of the bone marrow. Results represent mean Cd11b+/Ly6G+ cell numbers/ml from three mice per group. C: Immunofluorescence staining for neutrophils (red) and *C*. *albicans* (green), confirming absence of neutrophils in the tongues of mice receiving both 5Fu and C. albicans. D: Cytokine expression in tongue tissues of mice receiving 5Fu alone or with *C*. *albicans*. Interleukin-6 (IL-6), Keratinocyte derived cytokine (KC, IL-8) and Tumor Necrosis alpha (TNFa) protein concentrations were simultaneously quantified in tissue homogenates by multiplex ELISA, after standardizing protein content. The neutrophil activating cytokines IL-6 and KC were higher in the *C*. *albicans*-infected group. Bars represent average (± SD) fold increase over PBS-treated control group, with 5–8 mice/group. *p<0.0001, **p<0.001.
(TIF)

S2 Fig Bacterial burdens in tongue and small intestinal mucosal tissues. A: Linear regression plot of fungal and bacterial CFUs from the same tongues (n = 10). Mice were receiving 5Fu and *C*. *albicans* SC5314 in the drinking water for 8 days. A significant positive correlation was found between fungal and bacterial loads (R^2^ = 0.88, p<0.05). B: Tongue mucosa-associated bacterial loads in mice receiving 5Fu and *C*. *albicans* SC5314 daily in the drinking water. Mice were sacrificed 0, 2, 6 and 8 days later. Tongue was removed, weighed, homogenized, serially diluted and plated and results expressed as CFU counts/gm of tissue (left Y-axis, red dots). The total bacterial biomass (log 16S rRNA gene copy numbers/gm of tissue) was also quantified by real-time qPCR (right Y-axis, blue squares). Bacterial loads on day 8 differ significantly from untreated mice (day 0) and day 6 for CFUs (p = 0.009) and for log 16s copy numbers (p = 0.003). Data shown are from 2 independent mouse experiments, with 4–10 mice per group; bars represent means ± SEM. C: Small intestinal mucosa-associated bacterial loads in mice receiving 5Fu and *C*. *albicans* SC5314 daily in the drinking water. Mice were sacrificed 0, 2, 6 and 8 days later. The jejunum was removed, flushed with sterile PBS, weighed, homogenized, serially diluted and plated and results expressed as CFU counts/gm of tissue (left Y-axis, red dots). The total bacterial biomass (log 16S rRNA gene copy numbers/gm of tissue) was also quantified by real-time qPCR (right Y-axis, blue squares). Bacterial loads on day 8 did not differ significantly from untreated mice (day 0). Data shown are from 2 independent mouse experiments, with 4–10 mice per group; bars represent means ± SEM. D: Effect of 5Fu or *C*. *albicans* SC5314 (Ca) on cultivable intestinal bacterial counts. Mice were sacrificed on day 8 and tissues were collected and plated for CFUs as described above. Bacterial CFUs were compared to untreated control mice. 5Fu and *C*. *albicans* alone were associated with a statistically significant decrease in bacterial counts. Data shown are from 1–2 independent mouse experiments, with 4–5 mice per group; bars represent means ± SEM. *p<0.05, **p<0.01.
(TIF)

S3 Fig Bacterial microbiota profiling in tongue and small intestinal mucosal tissues. A: Observed OTU numbers (species) in each treatment group. Box plot showing average species numbers in untreated mice, mice receiving *C*. *albicans* SC5314 daily in the drinking water (Ca), mice receiving 5Fu alone and a combination of the two (5Fu+Ca) for 8 days. Mean values are shown from 5 mice in each group. In mice inoculated with *C*. *albicans* alone (Ca) bacterial diversity decreased in the tongue (p<0.01) but increased in the small intestinal mucosa (p<0.05), compared to untreated groups. For mice receiving 5Fu and *C*. *albicans* (5Fu+Ca) there was a significant further reduction in the number of species observed in tongue and small intestinal tissues (p<0.05 for tongue and p<0.01 for small intestine, compared to *C*. *albicans* alone). B: Time-dependent beta diversity changes based on Bray-Curtis dissimilarities among treatment groups. Shown are community structures in the two chemotherapy treatment groups (5Fu, red, 5Fu+Ca, blue; n = 5 mice/group) in tongues (triangles) and small intestines (squares) of the same mice. Early time points (days 2 and 6) are represented by open and day 8 by grey-filled shapes. Microbial community shifts in the 5Fu+Ca group clustered by timing of treatment, indicating that significant changes took place late in the infection process. In fact approximately 24% of the variability among these samples was explained by time of treatment (days 2 and 6 versus day 8, p<0.01). C: Mean relative abundance of OTU sequences assigned to one of the top 10 prominent taxa identified in small intestines, in each of the four treatment groups, at the end of the experimental period (day 8, n = 5 mice/group). *C*. *albicans* inoculation led to a significant relative abundance decrease in mucosa-associated lactobacilli whereas the dominant taxa in the group which also received 5Fu were *Alphaproteobacteria*, *Stenotrophomonas* and *Enterococcus*.
(TIF)

S4 Fig Effect of antibiotics on cultivable tongue bacteria. A: Cultivable bacterial counts in untreated mice, mice receiving antibiotics (Ant), mice receiving *C*. *albicans* SC5314 and antibiotics (Ca+Ant) and mice additionally receiving 5Fu (5Fu+Ca+Ant) for 8 days. The triple antibiotic regimen reduced cultivable bacterial counts to undetectable levels in all groups, with the exception of the group receiving 5Fu and *C*. *albicans*. Results shown are mean CFUs ± SEM from 5 mice/group. B: Identification of cultivable bacteria isolated from the tongues of two mice receiving 5Fu, *C*. *albicans* SC5314 and antibiotics at the end of the experimental period (day 8 post-infection). A total of five colonies were isolated from these mice and sequenced. As shown in this pie chart the dominant OTUs were *Staphylococcus* and *Stenotrophomonas*. No OTUs were identified aligning with the genus *Enterococcus*.
(TIF)

S5 Fig Epithelial permeability to FITC-dextran in a transwell assay. Confluent SCC15 cell monolayers seeded on the upper chamber were incubated with a PAR2 antagonist for 24 hours, followed by CCM from *E*. *faecalis* isolates for 16 hours. Permeability was measured by adding fluorescein isothiocyanate (FITC)-labeled dextran to the monolayers as described in methods. The flux of FITC-dextran across SCC15 cell monolayers (indicative of cellular permeability) was expressed as fold over untreated SCC15 cells. There was a significantly greater permeability when monolayers were apically treated with *E*. *faecalis* CCM and this was partly inhibited by a PAR2 inhibitor, suggesting a role of gelatinase E. Each experimental group represents 2 independent experiments with 3 technical replicates. *p<05, **p<0.0005.
(TIF)

## References

[1] Lalla RV, Latortue MC, Hong CH, Ariyawardana A, D'Amato-Palumbo S, Fischer DJ, Martof A, Nicolatou-Galitis O, Patton LL, Elting LS, Spijkervet FK, Brennan MT; Fungal Infections Section, Oral Care Study Group, Multinational Association of Supportive Care in Cancer (MASCC)/International Society of Oral Oncology (ISOO). A systematic review of oral fungal infections in patients receiving cancer therapy. Support Care Cancer. 2010 8;18(8):985–92. 10.1007/s00520-010-0892-z 20449755 PMC2914797

[2] Peterson DE, Boers-Doets CB, Bensadoun RJ, Herrstedt J; ESMO Guidelines Committee. Management of oral and gastrointestinal mucosal injury: ESMO Clinical Practice Guidelines for diagnosis, treatment, and follow-up. Ann Oncol. 2015 9;26 Suppl 5:v139–51. 10.1093/annonc/mdv202 Epub 2015 Jul 4. 26142468

[3] Cole GT, Halawa AA, Anaissie EJ. The role of the gastrointestinal tract in hematogenous candidiasis: from the laboratory to the bedside. Clinical infectious diseases: an official publication of the Infectious Diseases Society of America. 1996;22 Suppl 2:S73–88. 8722833 10.1093/clinids/22.supplement_2.s73

[4] Jarvis WR. Epidemiology of nosocomial fungal infections, with emphasis on Candida species. Clinical infectious diseases: an official publication of the Infectious Diseases Society of America. 1995;20(6):1526–30. 10.1093/clinids/20.6.1526 7548503

[5] Teoh F, Pavelka N. How Chemotherapy Increases the Risk of Systemic Candidiasis in Cancer Patients: Current Paradigm and Future Directions. Pathogens. 2016 1 15;5(1). pii: E6 10.3390/pathogens5010006 26784236 PMC4810127

[6] Ichim TE, Kesari S, Shafer K. Protection from chemotherapy- and antibiotic-mediated dysbiosis of the gut microbiota by a probiotic with digestive enzymes supplement. Oncotarget. 2018 7 20;9(56):30919–30935. 10.18632/oncotarget.25778 eCollection 2018 Jul 20. 30112118 PMC6089397

[7] Xu H, Dongari-Bagtzoglou A. Shaping the oral mycobiota: interactions of opportunistic fungi with oral bacteria and the host. Current opinion in microbiology. 2015;26:65–70. 10.1016/j.mib.2015.06.002 26100661 PMC4577367

[8] Xu H, Sobue T, Bertolini M, Thompson A, Dongari-Bagtzoglou A. Streptococcus oralis and Candida albicans Synergistically Activate mu-Calpain to Degrade E-cadherin From Oral Epithelial Junctions. The Journal of infectious diseases. 2016;214(6):925–34. 10.1093/infdis/jiw201 27190184 PMC4996146

[9] Xu H, Sobue T, Thompson A, Xie Z, Poon K, Ricker A, Cervantes J, Diaz PI, Dongari-Bagtzoglou A. Streptococcal co-infection augments Candida pathogenicity by amplifying the mucosal inflammatory response. Cellular microbiology. 2014;16(2):214–31. 10.1111/cmi.12216 24079976 PMC3956708

[10] Xu H, Sobue T, Bertolini M, Thompson A, Vickerman M, Nobile CJ, Dongari-Bagtzoglou A. S. oralis activates the Efg1 filamentation pathway in C. albicans to promote cross-kingdom interactions and mucosal biofilms. Virulence. 2017;8(8):1602–17. 10.1080/21505594.2017.1326438 28481721 PMC5810487

[11] Sobue T, Diaz P, Xu H, Bertolini M, Dongari-Bagtzoglou A. Experimental Models of C. albicans-Streptococcal Co-infection. Methods in molecular biology. 2016;1356:137–52. 10.1007/978-1-4939-3052-4_10 26519070

[12] Mason KL, Erb Downward JR, Falkowski NR, Young VB, Kao JY, Huffnagle GB. Interplay between the gastric bacterial microbiota and Candida albicans during postantibiotic recolonization and gastritis. Infection and immunity. 2012;80(1):150–8. 10.1128/IAI.05162-11 21986629 PMC3255670

[13] Mason KL, Erb Downward JR, Mason KD, Falkowski NR, Eaton KA, Kao JY, Young VB, Huffnagle GB. Candida albicans and bacterial microbiota interactions in the cecum during recolonization following broad-spectrum antibiotic therapy. Infection and immunity. 2012;80(10):3371–80. 10.1128/IAI.00449-12 22778094 PMC3457555

[14] Shankar J, Solis NV, Mounaud S, Szpakowski S, Liu H, Losada L, Nierman WC, Filler SG. Using Bayesian modelling to investigate factors governing antibiotic-induced Candida albicans colonization of the GI tract. Scientific reports. 2015;5:8131 10.1038/srep08131 25644850 PMC4314636

[15] Sandovsky-Losica H, Barr-Nea L, Segal E. Fatal systemic candidiasis of gastrointestinal origin: an experimental model in mice compromised by anti-cancer treatment. J Med Vet Mycol. 1992;30(3):219–31. 1517959 10.1080/02681219280000281

[16] Hata K, Horii T, Miyazaki M, Watanabe NA, Okubo M, Sonoda J, Nakamoto K, Tanaka K, Shirotori S, Murai N, Inoue S, Matsukura M, Abe S, Yoshimatsu K, Asada M. Efficacy of oral E1210, a new broad-spectrum antifungal with a novel mechanism of action, in murine models of candidiasis, aspergillosis, and fusariosis. Antimicrob Agents Chemother. 2011 10;55(10):4543–51. 10.1128/AAC.00366-11 Epub 2011 Jul 25. 21788462 PMC3187015

[17] Koh AY, Köhler JR, Coggshall KT, Van Rooijen N, Pier GB. Mucosal damage and neutropenia are required for Candida albicans dissemination. PLoS Pathog. 2008 2 8;4(2):e35 10.1371/journal.ppat.0040035 18282097 PMC2242836

[18] Clemons KV, Gonzalez GM, Singh G, Imai J, Espiritu M, Parmar R, Stevens DA. Development of an orogastrointestinal mucosal model of candidiasis with dissemination to visceral organs. Antimicrob Agents Chemother. 2006 8;50(8):2650–7. 10.1128/AAC.00530-06 16870754 PMC1538686

[19] Bertolini M, Sobue T, Thompson A, Dongari-Bagtzoglou A. Chemotherapy Induces Oral Mucositis in Mice Without Additional Noxious Stimuli. Transl Oncol. 2017 8;10(4):612–620. 10.1016/j.tranon.2017.05.001 Epub 2017 Jun 27. 28666190 PMC5491455

[20] Potting CM, Uitterhoeve R, Op Reimer WS, Van Achterberg T. The effectiveness of commonly used mouthwashes for the prevention of chemotherapy-induced oral mucositis: a systematic review. Eur J Cancer Care (Engl). 2006 12;15(5):431–9. 17177899 10.1111/j.1365-2354.2006.00684.x

[21] Altmeier S, Toska A, Sparber F, Teijeira A, Halin C, LeibundGut-Landmann S. IL-1 Coordinates the Neutrophil Response to C. albicans in the Oral Mucosa. PLoS Pathog. 2016 9 15;12(9):e1005882 10.1371/journal.ppat.1005882 eCollection 2016 Sep. 27632536 PMC5025078

[22] Madani TA. Clinical infections and bloodstream isolates associated with fever in patients undergoing chemotherapy for acute myeloid leukemia. Infection. 2000 Nov-Dec;28(6):367–73. 11139156 10.1007/s150100070007

[23] Mason KL, Erb Downward JR, Mason KD, Falkowski NR, Eaton KA, Kao JY, Young VB, Huffnagle GB. Candida albicans and bacterial microbiota interactions in the cecum during recolonization following broad-spectrum antibiotic therapy. Infect Immun. 2012 10;80(10):3371–80. 10.1128/IAI.00449-12 Epub 2012 Jul 9. 22778094 PMC3457555

[24] Rahman D, Mistry M, Thavaraj S, Challacombe SJ, Naglik JR. Murine model of concurrent oral and vaginal Candida albicans colonization to study epithelial host-pathogen interactions. Microbes Infect. 2007 4;9(5):615–22. Epub 2007 Jan 27. 10.1016/j.micinf.2007.01.012 17383212 PMC3242973

[25] Break TJ, Jaeger M, Solis NV, Filler SG, Rodriguez CA, Lim JK, Lee CC, Sobel JD, Netea MG, Lionakis MS. CX3CR1 is dispensable for control of mucosal Candida albicans infections in mice and humans. Infect Immun. 2015 3;83(3):958–65. 10.1128/IAI.02604-14 Epub 2014 Dec 29. 25547797 PMC4333470

[26] Sobue T, Bertolini M, Thompson A, Peterson DE, Diaz PI, Dongari-Bagtzoglou A. Chemotherapy-induced oral mucositis and associated infections in a novel organotypic model. Mol Oral Microbiol. 2018 6;33(3):212–223. 10.1111/omi.12214 Epub 2018 Feb 20. 29314782 PMC5945319

[27] Solis NV, Filler SG. Mouse model of oropharyngeal candidiasis. Nat Protoc. 2012 3;7(4):637–42. 10.1038/nprot.2012.011 22402633 PMC3671943

[28] Costa AC, Pereira CA, Junqueira JC, Jorge AO. Recent mouse and rat methods for the study of experimental oral candidiasis. Virulence. 2013 7 1;4(5):391–9. 10.4161/viru.25199 Epub 2013 May 28. 23715031 PMC3714131

[29] Villar CC, Kashleva H, Nobile CJ, Mitchell AP, Dongari-Bagtzoglou A. Mucosal tissue invasion by Candida albicans is associated with E-cadherin degradation, mediated by transcription factor Rim101p and protease Sap5p. Infect Immun. 2007 5;75(5):2126–35. Epub 2007 Mar 5. 10.1128/IAI.00054-07 17339363 PMC1865768

[30] Steck N, Hoffmann M, Sava IG, Kim SC, Hahne H, Tonkonogy SL, Mair K, Krueger D, Pruteanu M, Shanahan F, Vogelmann R, Schemann M, Kuster B, Sartor RB, Haller D. Enterococcus faecalis metalloprotease compromises epithelial barrier and contributes to intestinal inflammation. Gastroenterology. 2011 9;141(3):959–71. 10.1053/j.gastro.2011.05.035 21699778

[31] Maharshak N, Huh EY, Paiboonrungruang C, Shanahan M, Thurlow L, Herzog J, Djukic Z, Orlando R, Pawlinski R, Ellermann M, Borst L, Patel S, Dotan I, Sartor RB, Carroll IM. Enterococcus faecalis Gelatinase Mediates Intestinal Permeability via Protease-Activated Receptor 2. Infect Immun. 2015 7;83(7):2762–70. 10.1128/IAI.00425-15 Epub 2015 Apr 27. 25916983 PMC4468563

[32] Galloway-Peña JR, Bourgogne A, Qin X, Murray BE. Diversity of the fsr-gelE region of the Enterococcus faecalis genome but conservation in strains with partial deletions of the fsr operon. Appl Environ Microbiol. 2011 Jan;77(2):442–51. 10.1128/AEM.00756-10 Epub 2010 Nov 19. 21097591 PMC3020530

[33] Sawyer RT, Harmsen AG. The relative contribution of resident pulmonary alveolar macrophage and inflammatory polymorphonuclear neutrophils in host resistance to pulmonary infection by Candida albicans. Mycopathologia. 1989;108(2):95–105. 2687694 10.1007/BF00436059

[34] Polak-Wyss A. Protective effect of human granulocyte colony stimulating factor (hG-CSF) on Candida infections in normal and immunosuppressed mice. Mycoses. 1991;34(3–4):109–18. 1721105 10.1111/j.1439-0507.1991.tb00630.x

[35] Leendertse M, Willems RJ, Giebelen IA, Roelofs JJ, Bonten MJ, van der Poll T. Neutrophils are essential for rapid clearance of Enterococcus faecium in mice. Infect Immun. 2009 1;77(1):485–91. 10.1128/IAI.00863-08 Epub 2008 Nov 10. 19001080 PMC2612258

[36] Le Bastard Q, Ward T, Sidiropoulos D, Hillmann BM, Chun CL, Sadowsky MJ, Knights D, Montassier E. Fecal microbiota transplantation reverses antibiotic and chemotherapy-induced gut dysbiosis in mice. Sci Rep. 2018 4 18;8(1):6219 10.1038/s41598-018-24342-x PubMed 29670191 PMC5906603

[37] Sandovsky-Losica H, Segal E. Interaction of Candida albicans with murine gastrointestinal mucosa from methotrexate and 5-fluorouracil treated animals: in vitro adhesion and prevention. J Med Vet Mycol. 1990;28(4):279–87. 2269909

[38] Freedberg DE, Zhou MJ, Cohen ME, Annavajhala MK, Khan S, Moscoso DI, Brooks C, Whittier S, Chong DH, Uhlemann AC, Abrams JA. Pathogen colonization of the gastrointestinal microbiome at intensive care unit admission and risk for subsequent death or infection. Intensive care medicine. 2018 10.1007/s00134-018-5268-8 29936583 PMC6309661

[39] Bryant S, Wilbeck J. Vancomycin-resistant enterococcus in critical care areas. Critical care nursing clinics of North America. 2007;19(1):69–75. 10.1016/j.ccell.2006.10.005 17338952

[40] Jones DJ, Munro CL. Oral care and the risk of bloodstream infections in mechanically ventilated adults: A review. Intensive & critical care nursing. 2008;24(3):152–61. 10.1016/j.iccn.2008.01.004 18403205 PMC2753259

[41] Chomicz L, Szubinska D, Piekarczyk J, Wojtowicz A, Piekarczyk B, Starosciak B, Fiedor P. [Occurrence of oral subclinical infections in insulin treated diabetics]. Wiadomosci parazytologiczne. 2004;50(2):177–80. 16859022

[42] Komiyama EY, Lepesqueur LS, Yassuda CG, Samaranayake LP, Parahitiyawa NB, Balducci I, Koga-Ito CY. Enterococcus Species in the Oral Cavity: Prevalence, Virulence Factors and Antimicrobial Susceptibility. PloS one. 2016;11(9):e0163001 10.1371/journal.pone.0163001 27631785 PMC5025163

[43] Dahlen G, Blomqvist S, Almstahl A, Carlen A. Virulence factors and antibiotic susceptibility in enterococci isolated from oral mucosal and deep infections. Journal of oral microbiology. 2012;4 10.3402/jom.v4i0.10855 22368771 PMC3285953

[44] Jobbins J, Bagg J, Parsons K, Finlay I, Addy M, Newcombe RG. Oral carriage of yeasts, coliforms and staphylococci in patients with advanced malignant disease. Journal of oral pathology & medicine: official publication of the International Association of Oral Pathologists and the American Academy of Oral Pathology. 1992;21(7):305–8. 10.1111/j.1600-0714.1992.tb01016.x 1522531

[45] Holler E, Butzhammer P, Schmid K, Hundsrucker C, Koestler J, Peter K, Zhu W, Sporrer D, Hehlgans T, Kreutz M, Holler B, Wolff D, Edinger M, Andreesen R, Levine JE, Ferrara JL, Gessner A, Spang R, Oefner PJ. Metagenomic analysis of the stool microbiome in patients receiving allogeneic stem cell transplantation: loss of diversity is associated with use of systemic antibiotics and more pronounced in gastrointestinal graft-versus-host disease. Biology of blood and marrow transplantation: journal of the American Society for Blood and Marrow Transplantation. 2014;20(5):640–5. 10.1016/j.bbmt.2014.01.030 24492144 PMC4973578

[46] Dongari-Bagtzoglou A, Fidel PL Jr. The host cytokine responses and protective immunity in oropharyngeal candidiasis. Journal of dental research. 2005;84(11):966–77. 10.1177/154405910508401101 16246925

[47] Garsin DA, Lorenz MC. Candida albicans and Enterococcus faecalis in the gut: synergy in commensalism? Gut microbes. 2013;4(5):409–15. 10.4161/gmic.26040 23941906 PMC3839987

[48] Hermann C, Hermann J, Munzel U, Ruchel R. Bacterial flora accompanying Candida yeasts in clinical specimens. Mycoses. 1999;42(11–12):619–27. 10680438 10.1046/j.1439-0507.1999.00519.x

[49] Kovac J, Kovac D, Slobodnikova L, Kotulova D. Enterococcus faecalis and Candida albicans in the dental root canal and periapical infections. Bratislavske lekarske listy. 2013;114(12):716–20. 24329511

[50] Dahlen G, Blomqvist S, Almstahl A, Carlen A. Virulence factors and antibiotic susceptibility in enterococci isolated from oral mucosal and deep infections. Journal of oral microbiology. 2012;4 10.3402/jom.v4i0.10855 22368771 PMC3285953

[51] Clemons KV, Stevens DA. Orogastrointestinal model of mucosal and disseminated candidiasis. Methods Mol Biol. 2012;845:557–67. 10.1007/978-1-61779-539-8_41 22328404

[52] Wang H, Zhang W, Zuo L, Dong J, Zhu W, Li Y, Gu L, Gong J, Li Q, Li N, Li J. Intestinal dysbacteriosis contributes to decreased intestinal mucosal barrier function and increased bacterial translocation. Lett Appl Microbiol. 2014 4;58(4):384–92. 10.1111/lam.12201 Epub 2013 Dec 19. 24354719

[53] Jandhyala SM, Talukdar R, Subramanyam C, Vuyyuru H, Sasikala M, Nageshwar Reddy D. Role of the normal gut microbiota. World J Gastroenterol. 2015 8 7;21(29):8787–803. 10.3748/wjg.v21.i29.8787 26269668 PMC4528021

[54] Grabowska MM, Day ML. Soluble E-cadherin: more than a symptom of disease. Front Biosci (Landmark Ed). 2012 1 1;17:1948–64. 22201848 10.2741/4031 PMC4183062

[55] Samonte VA, Goto M, Ravindranath TM, Fazal N, Holloway VM, Goyal A, Gamelli RL, Sayeed MM. Exacerbation of intestinal permeability in rats after a two-hit injury: burn and Enterococcus faecalis infection. Crit Care Med. 2004 11;32(11):2267–73. 15640640 10.1097/01.ccm.0000145579.66001.05

[56] Ocvirk S, Sava IG, Lengfelder I, Lagkouvardos I, Steck N, Roh JH, Tchaptchet S, Bao Y, Hansen JJ, Huebner J, Carroll IM, Murray BE, Sartor RB, Haller D. Surface-Associated Lipoproteins Link Enterococcus faecalis Virulence to Colitogenic Activity in IL-10-Deficient Mice Independent of Their Expression Levels. PLoS Pathog. 2015 6 12;11(6):e1004911 10.1371/journal.ppat.1004911 eCollection 2015 Jun. 26067254 PMC4466351

[57] Kobayashi T, Glatz M, Horiuchi K, Kawasaki H, Akiyama H, Kaplan DH, Kong HH, Amagai M, Nagao K. Dysbiosis and Staphylococcus aureus Colonization Drives Inflammation in Atopic Dermatitis. Immunity. 2015 4 21;42(4):756–66. 10.1016/j.immuni.2015.03.014 25902485 PMC4407815

[58] Huycke MM, Abrams V, Moore DR. Enterococcus faecalis produces extracellular superoxide and hydrogen peroxide that damages colonic epithelial cell DNA. Carcinogenesis 23: 529–536, 2002 11895869 10.1093/carcin/23.3.529

[59] Ruiz PA, Shkoda A, Kim SC, Sartor RB, Haller D. IL-10 gene-deficient mice lack TGF-beta/Smad signaling and fail to inhibit proinflammatory gene expression in intestinal epithelial cells after the colonization with colitogenic Enterococcus faecalis. J Immunol. 2005 3 1;174(5):2990–9. 15728512 10.4049/jimmunol.174.5.2990

[60] Kurokawa K, Ryu KH, Ichikawa R, Masuda A, Kim MS, Lee H, Chae JH, Shimizu T, Saitoh T, Kuwano K, Akira S, Dohmae N, Nakayama H, Lee BL. Novel bacterial lipoprotein structures conserved in low-GC content gram-positive bacteria are recognized by Toll-like receptor 2. J Biol Chem. 2012 4 13;287(16):13170–81. 10.1074/jbc.M111.292235 Epub 2012 Feb 2. 22303020 PMC3339964

[61] Hoffmann M, Messlik A, Kim SC, Sartor RB, Haller D. Impact of a probiotic Enterococcus faecalis in a gnotobiotic mouse model of experimental colitis. Mol Nutr Food Res. 2011 5;55(5):703–13. 10.1002/mnfr.201000361 21254393

[62] Furrie E, Macfarlane S, Thomson G, Macfarlane GT; Microbiology & Gut Biology Group; Tayside Tissue & Tumour Bank. Toll-like receptors-2, -3 and -4 expression patterns on human colon and their regulation by mucosal-associated bacteria. Immunology. 2005 8;115(4):565–74. 10.1111/j.1365-2567.2005.02200.x 16011525 PMC1782176

[63] Gillum AM, Tsay EY, Kirsch DR. Isolation of the Candida albicans gene for orotidine-5'-phosphate decarboxylase by complementation of S. cerevisiae ura3 and E. coli pyrF mutations. Mol Gen Genet. 1984;198(2):179–82. 6394964 10.1007/BF00328721

[64] Villar CC, Kashleva H, Dongari-Bagtzoglou A. Role of Candida albicans polymorphism in interactions with oral epithelial cells. Oral Microbiol Immunol. 2004 8;19(4):262–9. 10.1111/j.1399-302X.2004.00150.x 15209998

[65] Meulendijks D, Cats A, Beijnen JH, Schellens JH. Improving safety of fluoropyrimidine chemotherapy by individualizing treatment based on dihydropyrimidine dehydrogenase activity—Ready for clinical practice? Cancer Treat Rev. 2016 11;50:23–34. 10.1016/j.ctrv.2016.08.002 27589829

[66] Cavalcanti IM, Nobbs AH, Ricomini-Filho AP, Jenkinson HF, Del Bel Cury AA. Interkingdom cooperation between Candida albicans, Streptococcus oralis and Actinomyces oris modulates early biofilm development on denture material. Pathog Dis. 2016 4;74(3). 10.1093/femspd/ftw002 26755532

[67] Pittman ME, Thomas BS, Wallace MA, Weber CJ, Burnham CA. Routine testing for anaerobic bacteria in cerebrospinal fluid cultures improves recovery of clinically significant pathogens. J Clin Microbiol. 2014 6;52(6):1824–9. 10.1128/JCM.00193-14 Epub 2014 Mar 12. 24622102 PMC4042729

[68] Abusleme L, Hong BY, Hoare A, Konkel JE, Diaz PI, Moutsopoulos NM. Oral Microbiome Characterization in Murine Models. Bio Protoc. 2017 12 20;7(24). 10.21769/BioProtoc.2655 PMC5760993 29333479

[69] Nadkarni MA, Martin FE, Jacques NA, Hunter N. Determination of bacterial load by real-time PCR using a broad-range (universal) probe and primers set. Microbiology. 2002 1;148(Pt 1):257–66 10.1099/00221287-148-1-257 11782518

[70] Ryu H, Henson M, Elk M, Toledo-Hernandez C, Griffith J, Blackwood D, Noble R, Gourmelon M, Glassmeyer S, Santo Domingo JW. Development of quantitative PCR assays targeting the 16S rRNA genes of Enterococcus spp. and their application to the identification of enterococcus species in environmental samples. Appl Environ Microbiol. 2013 1;79(1):196–204 10.1128/AEM.02802-12 23087032 PMC3536114

[71] Caporaso JG, Lauber CL, Walters WA, Berg-Lyons D, Huntley J, Fierer N, Owens SM, Betley J, Fraser L, Bauer M, Gormley N, Gilbert JA, Smith G, Knight R. Ultra-high-throughput microbial community analysis on the Illumina HiSeq and MiSeq platforms. ISME J. 2012 8;6(8):1621–4. 10.1038/ismej.2012.8 22402401 PMC3400413

[72] Kozich JJ, Westcott SL, Baxter NT, Highlander SK, Schloss PD. Development of a dual-index sequencing strategy and curation pipeline for analyzing amplicon sequence data on the MiSeq Illumina sequencing platform. Appl Environ Microbiol. 2013 9;79(17):5112–20. 10.1128/AEM.01043-13 Epub 2013 Jun 21. 23793624 PMC3753973

[73] Wang Q, Garrity GM, Tiedje JM, Cole JR. Naive Bayesian classifier for rapid assignment of rRNA sequences into the new bacterial taxonomy. Appl Environ Microbiol. 2007 8;73(16):5261–7. Epub 2007 Jun 22. 10.1128/AEM.00062-07 17586664 PMC1950982

[74] Jackson CR, Fedorka-Cray PJ, Barrett JB. Use of a genus- and species-specific multiplex PCR for identification of enterococci. J Clin Microbiol. 2004 8;42(8):3558–65. 10.1128/JCM.42.8.3558-3565.2004 15297497 PMC497640

[75] Dongari-Bagtzoglou A, Kashleva H, Dwivedi P, Diaz P, Vasilakos J. Characterization of mucosal Candida albicans biofilms. PLoS One. 2009 11 24;4(11):e7967 10.1371/journal.pone.0007967 19956771 PMC2776351

[76] Amann RI, Binder BJ, Olson RJ, Chisholm SW, Devereux R, et al Combination of 16S ribosomal RNA-targeted oligonucleotide probes with flow cytometry for analyzing mixed microbial populations. Appl Env Microbiol 1990; 56: 1919–25. 2200342 10.1128/aem.56.6.1919-1925.1990 PMC184531

[77] Waar K, Degener JE, van Luyn MJ, Harmsen HJ. Fluorescent in situ hybridization with specific DNA probes offers adequate detection of Enterococcus faecalis and Enterococcus faecium in clinical samples. J Med Microbiol. 2005 10;54(Pt10):937–44. 16157547 10.1099/jmm.0.46022-0

[78] Dongari-Bagtzoglou A, Kashleva H. Development of a highly reproducible three-dimensional organotypic model of the oral mucosa. Nat Protoc. 2006;1(4):2012–8. 10.1038/nprot.2006.323 17487190 PMC2699620

[79] Ruiz-Cruz S, Espinosa M, Goldmann O, Bravo A. Global Regulation of Gene Expression by the MafR Protein of Enterococcus faecalis. Front Microbiol. 2016 1 11;6:1521 10.3389/fmicb.2015.01521 eCollection 2015. 26793169 PMC4707282

[80] Shepard BD, Gilmore MS. Differential expression of virulence-related genes in Enterococcus faecalis in response to biological cues in serum and urine. Infect Immun. 2002 8;70(8):4344–52. 10.1128/IAI.70.8.4344-4352.2002 12117944 PMC128128

