# Supplementary figures and images for: Candida albicans induces mucosal bacterial dysbiosis that promotes invasive infection
Source: PLoS Pathog. 2019 Apr 22;15(4):e1007717. doi: 10.1371/journal.ppat.1007717 (PMC6497318; doi:10.1371/journal.ppat.1007717)

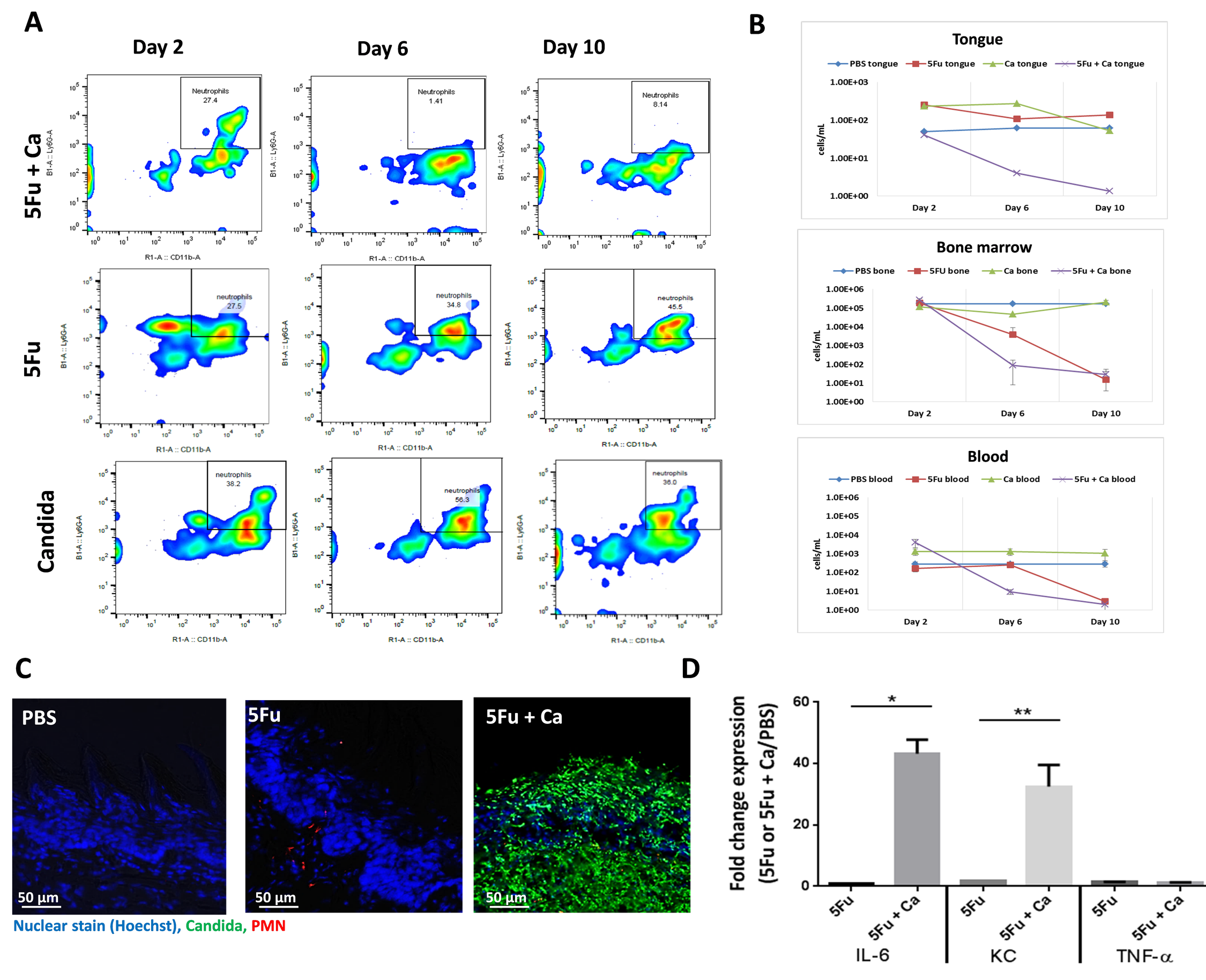

Supplement: S1 Fig — A: Time-dependent analysis of tongue neutrophils. Mice receiving 5Fu (50mg/kg, IV, every 48 hours) + C. albicans (5Fu+Ca), and either 5Fu or Candida alone were sacrificed at the indicated time points and CD11b+/Ly6G+ cells extracted from tongues were sorted by FACS. Note the almost complete depletion of tongue neutrophils in the 5Fu+Ca group by day 6. B: Time-dependent analysis of neutrophil numbers in tongue, bone marrow and peripheral blood. In tongue tissues the 5Fu and Candida alone groups had a steady influx of neutrophils, slightly higher than the PBS control on days 2 and 6, whereas a dramatic drop was noted in the 5Fu+Candida group by day 6. In the bone marrow mature cells declined rapidly in both groups receiving 5Fu, consistent with the myelosuppressive action of this drug. Blood counts paralleled those of the bone marrow. Results represent mean Cd11b+/Ly6G+ cell numbers/ml from three mice per group. C: Immunofluorescence staining for neutrophils (red) and C. albicans (green), confirming absence of neutrophils in the tongues of mice receiving both 5Fu and C. albicans. D: Cytokine expression in tongue tissues of mice receiving 5Fu alone or with C. albicans. Interleukin-6 (IL-6), Keratinocyte derived cytokine (KC, IL-8) and Tumor Necrosis alpha (TNFa) protein concentrations were simultaneously quantified in tissue homogenates by multiplex ELISA, after standardizing protein content. The neutrophil activating cytokines IL-6 and KC were higher in the C. albicans-infected group. Bars represent average (± SD) fold increase over PBS-treated control group, with 5–8 mice/group. *p<0.0001, **p<0.001. (TIF) [file ppat.1007717.s001.tif]

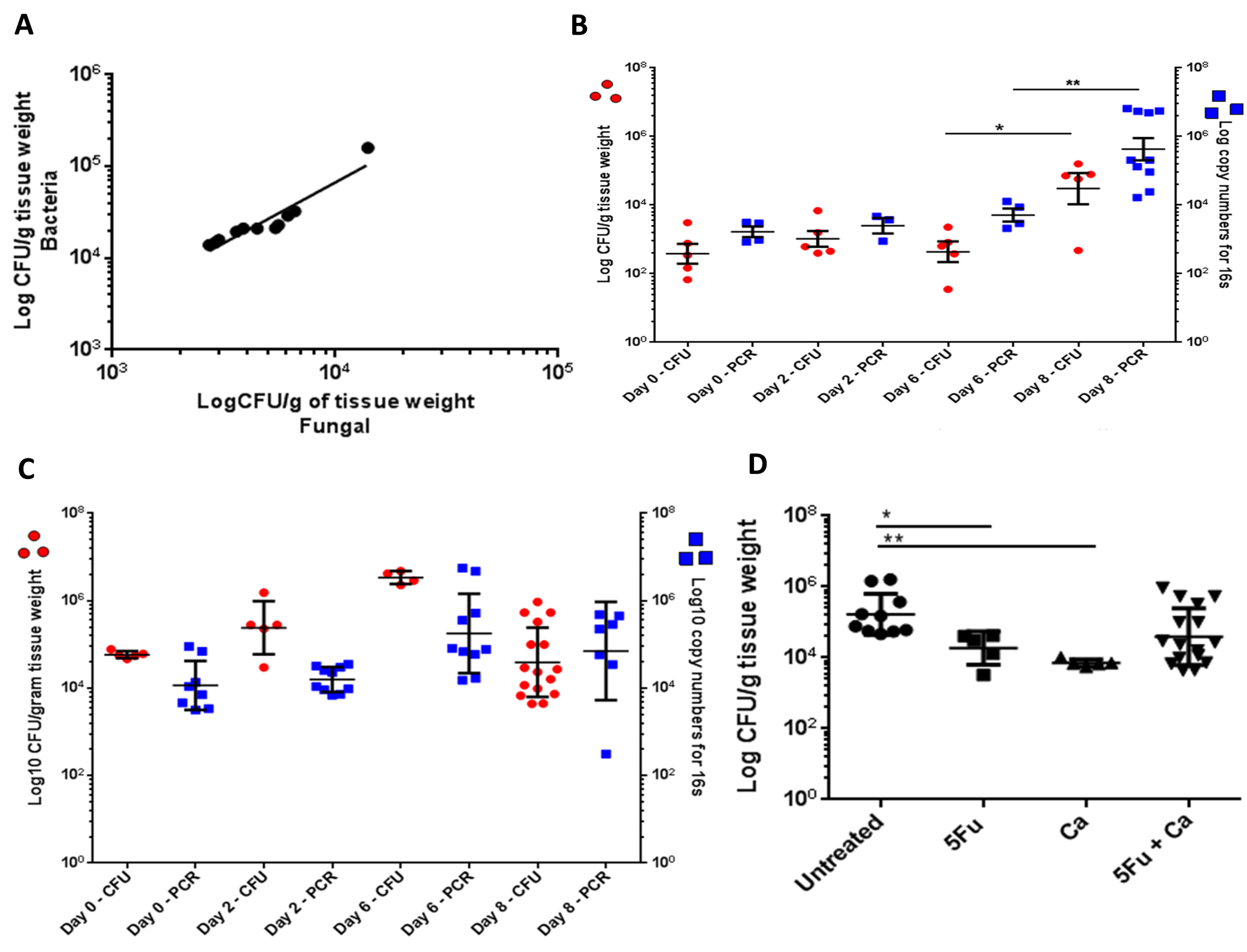

Supplement: S2 Fig — A: Linear regression plot of fungal and bacterial CFUs from the same tongues (n = 10). Mice were receiving 5Fu and C. albicans SC5314 in the drinking water for 8 days. A significant positive correlation was found between fungal and bacterial loads (R2 = 0.88, p<0.05). B: Tongue mucosa-associated bacterial loads in mice receiving 5Fu and C. albicans SC5314 daily in the drinking water. Mice were sacrificed 0, 2, 6 and 8 days later. Tongue was removed, weighed, homogenized, serially diluted and plated and results expressed as CFU counts/gm of tissue (left Y-axis, red dots). The total bacterial biomass (log 16S rRNA gene copy numbers/gm of tissue) was also quantified by real-time qPCR (right Y-axis, blue squares). Bacterial loads on day 8 differ significantly from untreated mice (day 0) and day 6 for CFUs (p = 0.009) and for log 16s copy numbers (p = 0.003). Data shown are from 2 independent mouse experiments, with 4–10 mice per group; bars represent means ± SEM. C: Small intestinal mucosa-associated bacterial loads in mice receiving 5Fu and C. albicans SC5314 daily in the drinking water. Mice were sacrificed 0, 2, 6 and 8 days later. The jejunum was removed, flushed with sterile PBS, weighed, homogenized, serially diluted and plated and results expressed as CFU counts/gm of tissue (left Y-axis, red dots). The total bacterial biomass (log 16S rRNA gene copy numbers/gm of tissue) was also quantified by real-time qPCR (right Y-axis, blue squares). Bacterial loads on day 8 did not differ significantly from untreated mice (day 0). Data shown are from 2 independent mouse experiments, with 4–10 mice per group; bars represent means ± SEM. D: Effect of 5Fu or C. albicans SC5314 (Ca) on cultivable intestinal bacterial counts. Mice were sacrificed on day 8 and tissues were collected and plated for CFUs as described above. Bacterial CFUs were compared to untreated control mice. 5Fu and C. albicans alone were associated with a statistically significant decrease in bacterial counts. [file ppat.1007717.s002.tif]

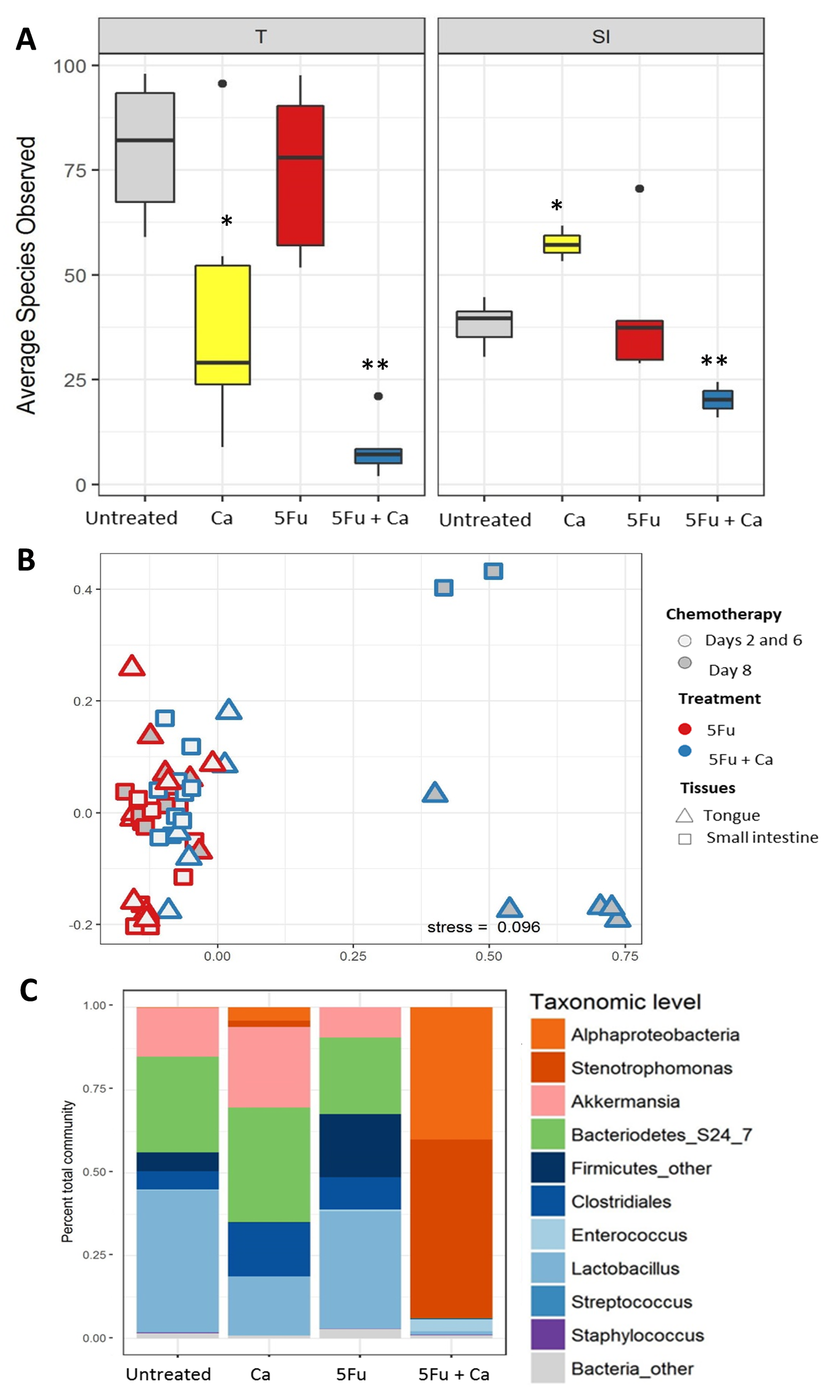

Supplement: S3 Fig — A: Observed OTU numbers (species) in each treatment group. Box plot showing average species numbers in untreated mice, mice receiving C. albicans SC5314 daily in the drinking water (Ca), mice receiving 5Fu alone and a combination of the two (5Fu+Ca) for 8 days. Mean values are shown from 5 mice in each group. In mice inoculated with C. albicans alone (Ca) bacterial diversity decreased in the tongue (p<0.01) but increased in the small intestinal mucosa (p<0.05), compared to untreated groups. For mice receiving 5Fu and C. albicans (5Fu+Ca) there was a significant further reduction in the number of species observed in tongue and small intestinal tissues (p<0.05 for tongue and p<0.01 for small intestine, compared to C. albicans alone). B: Time-dependent beta diversity changes based on Bray-Curtis dissimilarities among treatment groups. Shown are community structures in the two chemotherapy treatment groups (5Fu, red, 5Fu+Ca, blue; n = 5 mice/group) in tongues (triangles) and small intestines (squares) of the same mice. Early time points (days 2 and 6) are represented by open and day 8 by grey-filled shapes. Microbial community shifts in the 5Fu+Ca group clustered by timing of treatment, indicating that significant changes took place late in the infection process. In fact approximately 24% of the variability among these samples was explained by time of treatment (days 2 and 6 versus day 8, p<0.01). C: Mean relative abundance of OTU sequences assigned to one of the top 10 prominent taxa identified in small intestines, in each of the four treatment groups, at the end of the experimental period (day 8, n = 5 mice/group). C. albicans inoculation led to a significant relative abundance decrease in mucosa-associated lactobacilli whereas the dominant taxa in the group which also received 5Fu were Alphaproteobacteria, Stenotrophomonas and Enterococcus. (TIF) [file ppat.1007717.s003.tif]

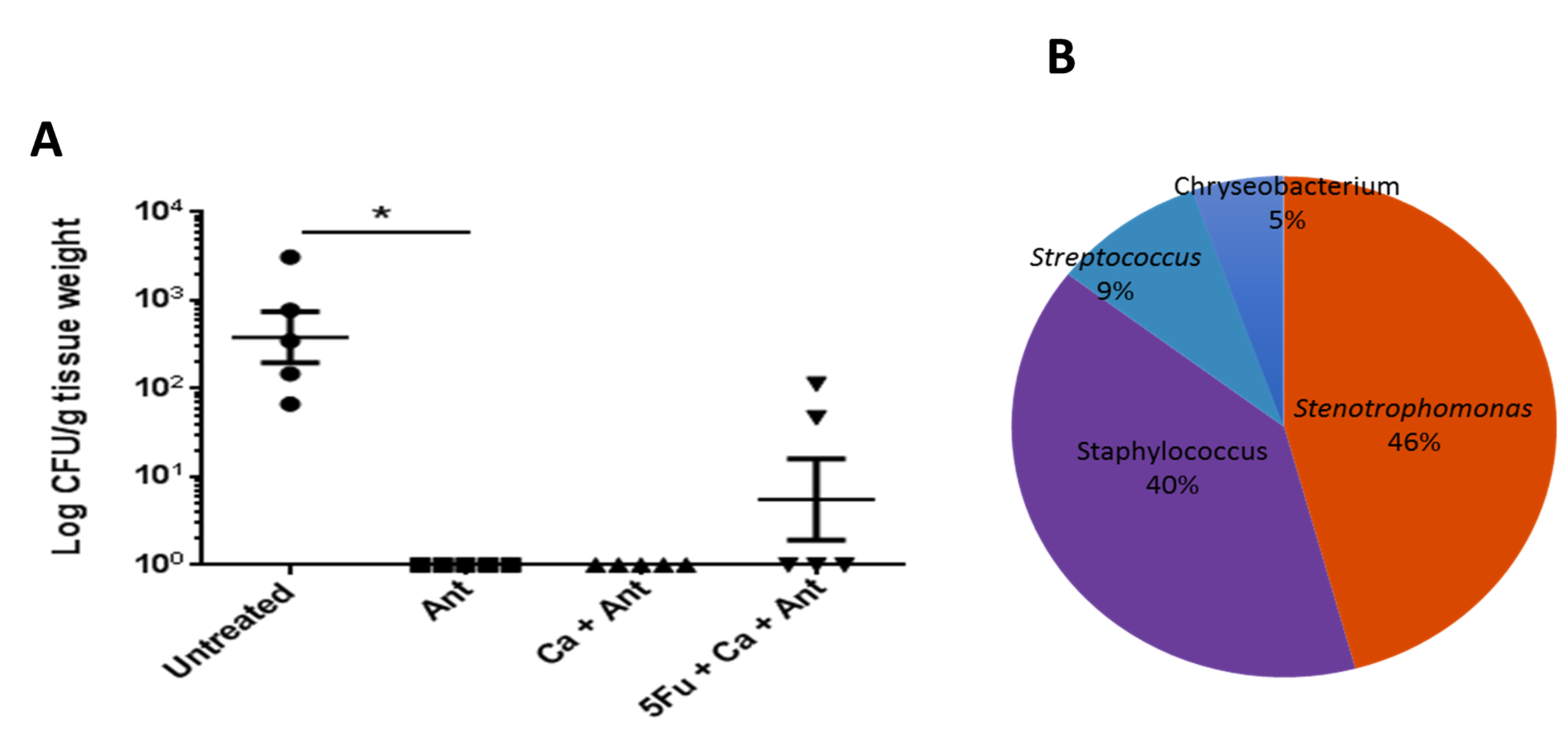

Supplement: S4 Fig — A: Cultivable bacterial counts in untreated mice, mice receiving antibiotics (Ant), mice receiving C. albicans SC5314 and antibiotics (Ca+Ant) and mice additionally receiving 5Fu (5Fu+Ca+Ant) for 8 days. The triple antibiotic regimen reduced cultivable bacterial counts to undetectable levels in all groups, with the exception of the group receiving 5Fu and C. albicans. Results shown are mean CFUs ± SEM from 5 mice/group. B: Identification of cultivable bacteria isolated from the tongues of two mice receiving 5Fu, C. albicans SC5314 and antibiotics at the end of the experimental period (day 8 post-infection). A total of five colonies were isolated from these mice and sequenced. As shown in this pie chart the dominant OTUs were Staphylococcus and Stenotrophomonas. No OTUs were identified aligning with the genus Enterococcus. (TIF) [file ppat.1007717.s004.tif]

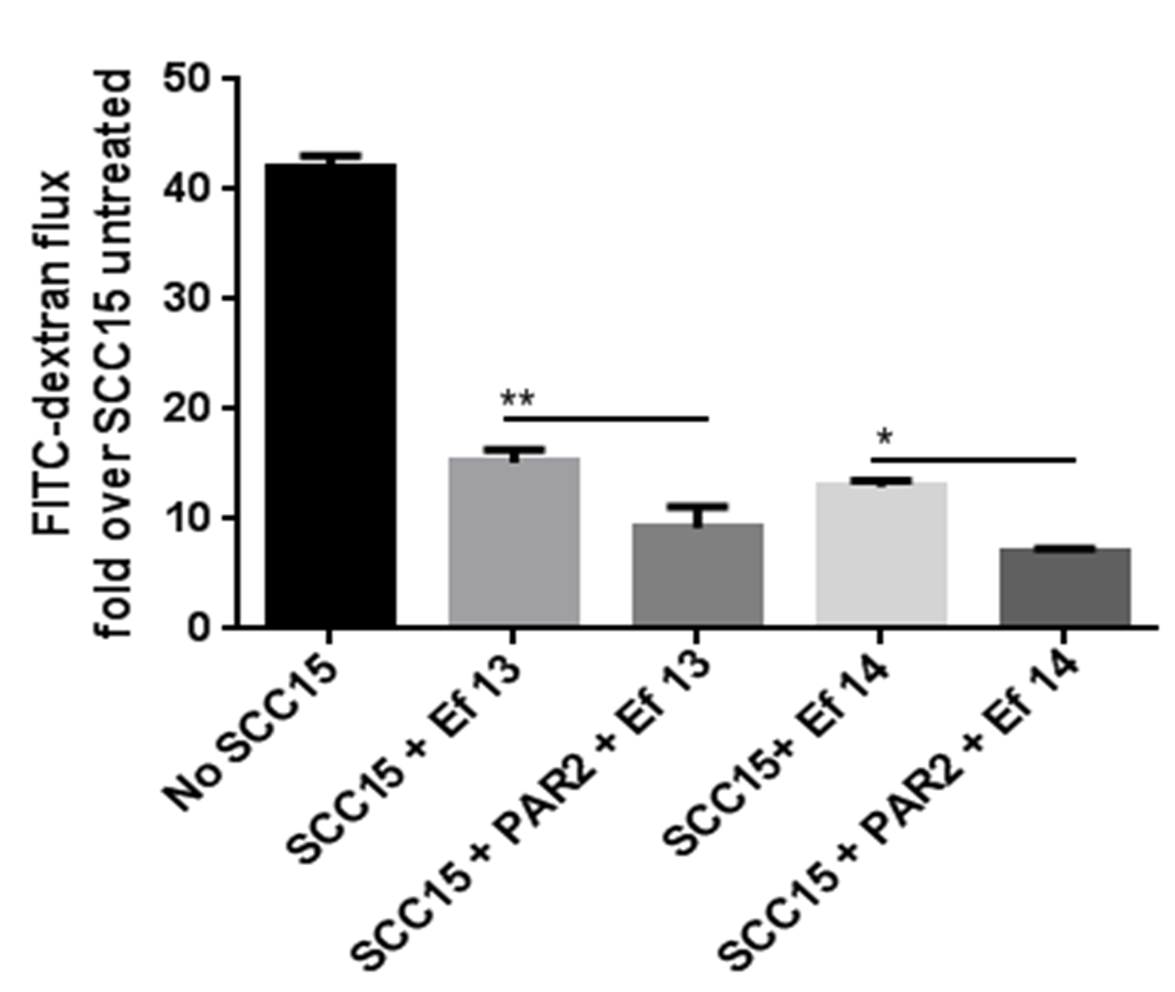

Supplement: S5 Fig — Confluent SCC15 cell monolayers seeded on the upper chamber were incubated with a PAR2 antagonist for 24 hours, followed by CCM from E. faecalis isolates for 16 hours. Permeability was measured by adding fluorescein isothiocyanate (FITC)-labeled dextran to the monolayers as described in methods. The flux of FITC-dextran across SCC15 cell monolayers (indicative of cellular permeability) was expressed as fold over untreated SCC15 cells. There was a significantly greater permeability when monolayers were apically treated with E. faecalis CCM and this was partly inhibited by a PAR2 inhibitor, suggesting a role of gelatinase E. Each experimental group represents 2 independent experiments with 3 technical replicates. *p<05, **p<0.0005. (TIF) [file ppat.1007717.s005.tif]
